# Long-term retention of antigens in germinal centres is controlled by the spatial organisation of the follicular dendritic cell network

**DOI:** 10.1038/s41590-023-01559-1

**Published:** 2023-07-13

**Authors:** Ana Martínez-Riaño, Shenshen Wang, Stefan Boeing, Sophie Minoughan, Antonio Casal, Katelyn M Spillane, Burkhard Ludewig, Pavel Tolar

**Affiliations:** 1 Immune Receptor Activation Laboratory, The Francis Crick Institute, London, UK; 2 Division of Infection and Immunity, Institute of Immunity and Transplantation, University College London, London, UK; 3 Department of Physics and Astronomy, University of California Los Angeles, Los Angeles, CA, USA; 4 Bioinformatics and Biostatistics Science Technology Platform, The Francis Crick Institute, UK; 5 Department of Physics, King’s College London, London, UK; 6 Randall Centre for Cell and Molecular Biophysics, King’s College London, UK; 7 Institute of Immunobiology, Medical Research Center, Kantonsspital St. Gallen, St. Gallen, Switzerland

## Abstract

Germinal centers (GCs) require sustained availability of antigens to promote antibody affinity maturation against pathogens and vaccines. A key source of antigens for GC B cells are immune complexes (ICs) displayed on follicular dendritic cells (FDCs). Here we show that FDC spatial organization regulates antigen dynamics in the GC. We identify the existence of a broader FDC heterogeneity within the network. While the entire light zone (LZ) FDC network captures ICs initially, only the central cells of the network function as the antigen reservoir, where different antigens arriving from subsequent immunizations co-localize. Mechanistically, central LZ FDCs constitutively express subtly higher CR2 membrane densities than peripheral LZ FDCs, which strongly increases the IC retention half-life. Even though repeated immunizations gradually saturate central FDCs, B cell responses remain efficient because new antigens partially displace old ones. These results reveal the principles shaping antigen display on FDCs during the GC reaction.

## Introduction

The generation of high-affinity antibodies that neutralize pathogens is a hallmark of the humoral response. The response is initiated by the binding of antigens to antigen-specific B cells in the follicles of secondary lymphoid organs. Activated B cells generate an initial burst of plasma and memory cells and also seed germinal centers (GCs), where they further diversify their B cell receptor (BCR) repertoire and undergo selection for antigen affinity^1^. B cells with higher affinity BCRs outcompete B cells with lower affinity thanks to the survival and proliferative advantage instigated by higher BCR binding to antigens and higher T cell help^2,3^. This selection takes several weeks and increases the affinity of antibodies produced by GC-generated plasma cells (PCs). Thus, a constant supply of antigens to GC B cells is essential to fuel affinity maturation, critical for antibody-mediated protection.

Although secondary lymphoid organs are constantly exposed to antigens in lymph or blood, the entry of antigens into B cell follicles is restricted^4,5^ and their retention require binding to follicular dendritic cells (FDCs)^6–8^. FDCs are stromal cells that develop from perivascular or subcapsular precursors of the spleen and lymph node, respectively,^9,10^ in response to LTα1β2 and TNF produced by B cells^11–14^. FDCs form tight networks throughout the follicle via long intermingled dendrites and produce factors that control B cell survival, localization and elimination upon apoptosis (e.g., CXCL13^15^, GGT5^16^, TNFSF13B (BAFF)^17^ and MFGE8^18^). Within the first day after immunization, complement-coated antigens are shuttled to FDCs by non-cognate B cells from the subcapsular sinus of the LN or from the marginal zone of the spleen^19,20^. Subsequently, FDCs retain and display the intact antigens for several weeks. A related subset termed dark zone (DZ) FDC (also called *Cxcl12*-expressing reticular cells, CRCs) does not retain antigens but organizes the GC DZ by producing CXCL12^6,21^.

Canonical (also termed light zone, LZ) FDCs capture antigens in complement-coated particles or immune complexes (ICs) via two complement receptors, CR1 (CD35) and CR2 (CD21), which bind to the complement fragment C3d covalently attached to the antigen. In the mouse, both receptors are encoded by a single *Cr2* gene and we, therefore, refer to them both as CR2 here. In addition, a subpopulation of activated FDCs in the GC expresses an array of Fc-receptors (FCGR2B, FCER2A and FCMAR)^22^, whose roles in antigen retention are less clear, but may fine-tune B cell selection^23–26^.

In line with the ability of FDCs to retain antigens, the deletion of CR2 from stromal cells reduces antibody responses to primary and secondary immunization^27^. However, some studies did not find a role for FDC antigen retention in the GC response^28^. Nevertheless, enhancing the deposition of antigens on FDCs leads to augmented GC responses and the production of highly evolved antibody specificities, suggesting that targeting antigens to FDCs may be beneficial in vaccine-induced protection^29–31^.

To better understand the long-term dynamics of antigen retention in B cell follicles and their replacement upon reimmunization, we imaged clarified LNs from mice immunized with fluorescent ICs. We observed striking changes in antigen localization over time: while the entire FDC network captured antigen initially, only the central cells retained antigen throughout the duration of the GC and served as long-term antigen reservoirs. This pattern was independent of the GC itself. Single-cell transcriptomics corroborated functional heterogeneity within LZ FDCs. Mechanistically, central FDCs had slower IC dissociation due to subtly higher levels of CR2 on their surfaces. Repeated immunizations partially saturated the central FDCs, but also replaced previous antigens, suggesting a dynamic competition of ICs for CR2. Antigen replacement on central FDCs, together with non-saturable antigen capture by peripheral FDCs, may underlie the efficient B cell response to repeated challenges. Understanding the basis of antigen dynamics on the FDC network will guide the generation of more efficient vaccines aiming to improve antigen retention in the GC.

## Results

### Long-term antigen retention is mediated by central FDCs

**Extended Data Figure 1:**
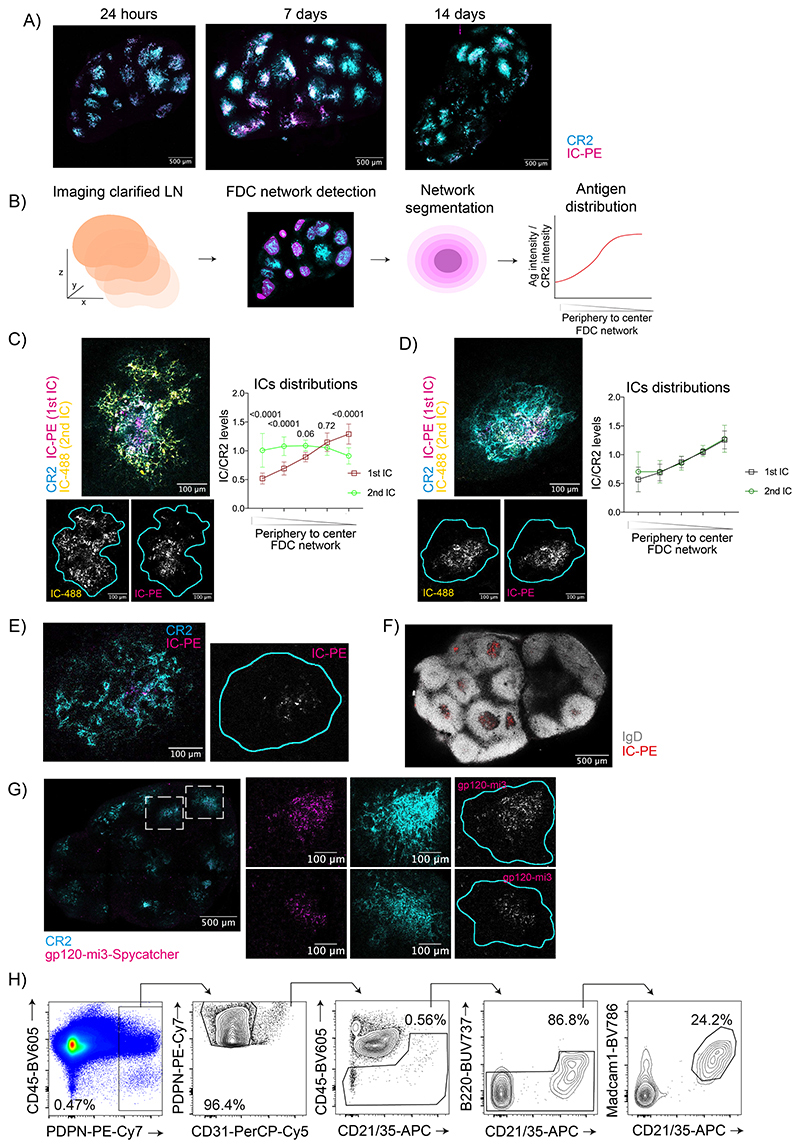
Antigens centralize on the FDC network. A) Maximum intensity projection of confocal images of clarified LNs of mice immunized with IC-PE (magenta). FDC networks are in cyan (anti-CD21/35) (n = 6 LNs; 3 experiments). B) Image analysis to quantify antigen distribution within each B cell follicle. C) Confocal image of an FDC network (cyan) after immunization with two subsequent ICs in PBS analyzed 7 days after the first immunization (IC-PE; magenta) and 24 hours after the second (IC-488; yellow). Single-color images of IC-488 (left) and IC-PE (right) are shown below. Cyan line demarcates FDC network boundary based on anti-CD21/35 staining. Right, quantification of the distribution of both antigens on the FDC network. (n= 8 LNs; 2 experiments). D) Confocal image of an FDC network (cyan) after immunization with two subsequent ICs as in C for 14 and 7 days. Single-color images of IC-488 (left) and IC-PE (right) are shown below. Cyan line demarcates the FDC network boundary based on anti-CD21/35 staining. Right, quantification of the distribution of both antigens on the FDC network (n = 12 LNs; 2 experiments). E) Image of an LN FDC network (cyan) 56 days after immunization with IC-PE (magenta). Right, single-color image of IC-PE (grey) with cyan line demarcating the FDC network boundary based on anti-CD21/35 staining (n = 4 LNs; 1 experiment). F) Image of a draining LN 21 days after immunization with IC-PE (red). Naïve B cells are shown in grey (anti-IgD). (n=4 LNs; 2 experiments). G) Image of a draining LN 7 days after immunization with AF555-labelled mi3-Spycatcher nanoparticles coated with YU-gp120-Spytag HIV envelope protein (magenta). FDC networks are shown in cyan (anti-CD21/35). White square indicates the region magnified. Cyan line demarcating the FDC network boundary based on anti-CD21/35 staining (n = 3 LNS; 2 experiments). H) Flow cytometry gating strategy to analyze FDCs. Quantitative data show the mean ± SD analysis by two-tailed t-test or one-way ANOVA with multiple comparisons.

**Figure 1:**
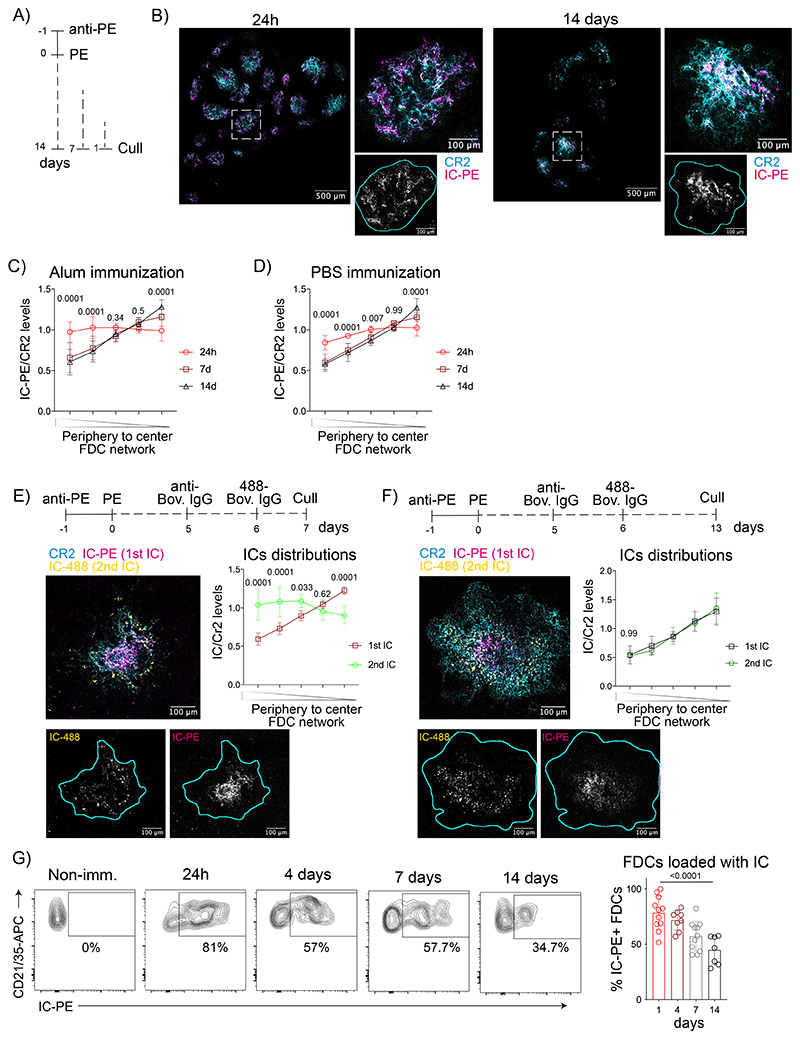
Long-term dynamics of antigen localization in B cell follicles. A) Immunization workflow to analyze IC-PE localization in draining LNs after 24 hours, 7 and 14 days. IC-PE immunization is performed by injecting PE-specific antibody (anti-PE) i. p. followed by PE s.c. the day after. B) Representative confocal section of clarified draining LNs 24 hours (left) and 14 days (right) after immunization with IC-PE (magenta). FDC networks are stained by anti-CD21/35 antibody binding to CR2 on FDCs (cyan). White squares indicate the region magnified. Single-color image of IC-PE is shown in grey. Cyan line demarcates the FDC network boundary based on anti-CD21/35 staining. C) Quantification of antigen distribution on the FDC networks of mice immunized with IC-PE for 24 hours (n=6), 7 (n=8) and 14 days (n=9) with antigen embedded in Alum. Quantification workflow is described in Extended Data Figure 1A (3 experiments). D) Antigen distribution on the FDC network of mice immunized as described in (C) with antigen diluted in PBS (n=6 and 7 mice; 2 experiments). E) Distribution of ICs on the FDC network after a repeated immunization. LNs were analyzed 7 days after the first IC injection (IC-PE) and 24 hours after the second one (IC-488). A representative confocal image of an FDC network (anti-CD21/35 in cyan; IC-PE in magenta; IC-488 in yellow) with single-color images of IC-488 (left) and IC-PE (right) shown below. Cyan line demarcates the FDC network boundary based on anti-CD21/35 staining. Quantification of the distribution of both ICs in the FDC network is shown in the graph (n = 6 mice; 2 experiments) F) Distribution of ICs on the FDC network 7 days after the repeated immunization (IC488, 2^nd^ IC) (n=5 mice) and 14 days (IC-PE, 1^st^ IC) (n=7 mice) after the first immunization as described for (E) (2 experiments). G) Flow cytometry plots and quantification of the percentage of IC-PE^+^ FDCs at different time-points after IC-PE immunization (n = 7, 8 and11 mice). All quantitative data show means ± SD and analysis by two-tailed two-way’s ANOVA with multiple comparisons.

To study the retention of vaccine antigens during the GC response, we immunized mice with fluorescent antigen immune complexes (IC), known to efficiently bind to FDCs^19^, and imaged clarified draining lymph nodes (LN) at different time-points post-injection using 3D confocal microscopy (Figure 1A). We observed that 24 hours postimmunization, most of the antigen was loaded onto the entire FDC network of each B cell follicle. However, on days 7 and 14 after immunization, when GCs form, the antigen localization became restricted to the center of each follicle (Figure 1B and Extended Data Figure 1A). To quantify the distribution of the antigen over time, we divided each FDC network into five concentric shells and calculated the ratio of antigen to anti-CR2 fluorescence (Extended Data Figure 1B). The quantification confirmed that on day 1 postimmunization, antigen was distributed equally across the FDC networks, while on days 7 and 14 after immunization, it was preferentially located in the center (Figure 1C). This antigen centralization was independent of the presence of the alum adjuvant during immunization (Figure 1C, D). Thus, antigen localization is dynamic during the onset of the GC.

To understand if subsequent immunizations generate a similar pattern of antigen distribution, we followed the first immunization (IC-PE) 6 days later with a second immunization with a different antigen-IC (IC-488; Figure 1E and F, Extended Data Figure 1C, D). Imaging of clarified LNs showed that 24 hours after the second immunization, the second antigen-IC was present over the entire FDC network, while the first antigen was already localized in the center. Seven days later, the second antigen also centralized and co-localized with the first antigen on the central FDCs. Thus, while the entire FDC network captures incoming ICs initially, the retention of the antigens takes place exclusively on the central FDCs of the network, independently of the presence of previous antigens.

We observed antigen retention on central FDCs up to 56 days post-immunization (Extended Data Figure 1E). On day 21, the centralized antigen co-localized with GCs (Extended Data Figure 1F). Similar localization of antigens on central FDCs was observed 7 days after immunization with HIV-gp120 nanoparticles (Extended Data Figure 1G), which trigger complement activation independently of IC formation^24^. Furthermore, flow cytometry showed that the percentage of FDCs loaded with antigen-IC decreased from day 1 to day 14 postimmunization (Extended Data Figure 1H, Figure 1G). Altogether, these data suggest a heterogeneity within the FDC network, with the periphery capturing antigens transiently and the center retaining antigens beyond the first-week post-immunization.

### Dynamics of antigen retention are independent of the GC

**Extended Data Figure 2:**
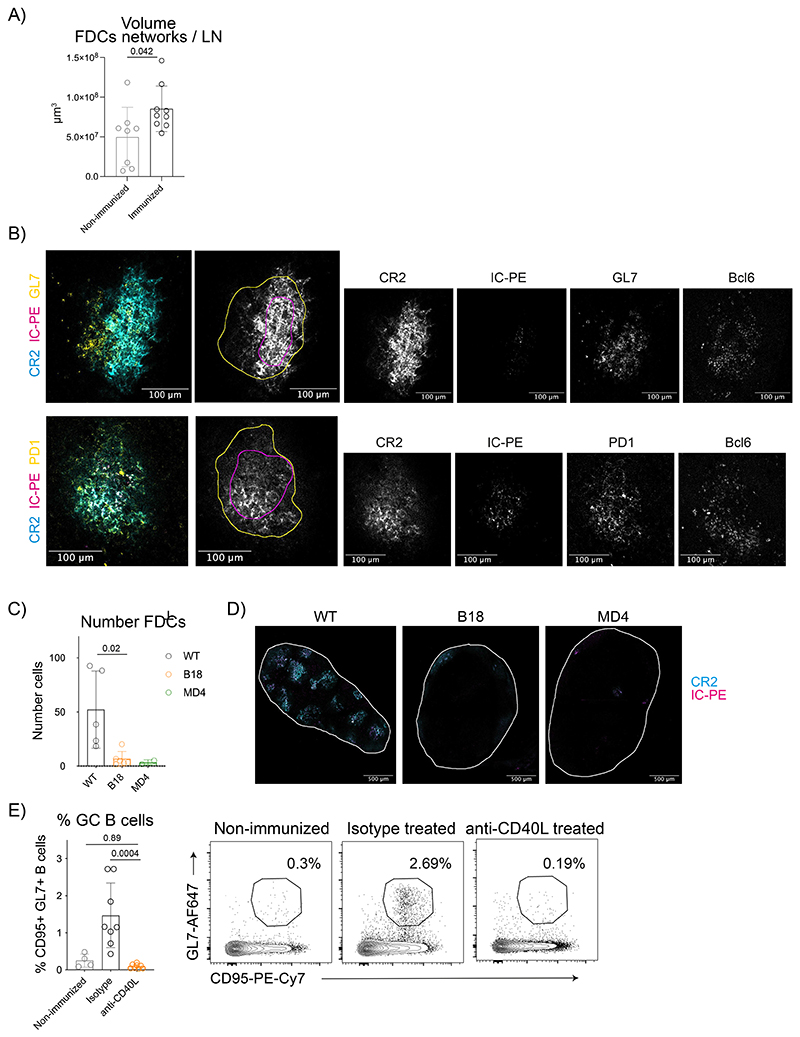
B cell activation is required for FDC expansion. A) Quantification of the FDC network volume per LN based on anti-CD21/35 staining in clarified LNs from non-immunized (n = 8 mice) and 13 days post-immunized (n = 9) mice with IC-PE. B) Representative immunofluorescence images of LN B cell GCs from mice immunized for 14 days with IC-PE. Upper row shows the merged image of GL7 (yellow), IC-PE (magenta), and anti-CD21/35 (cyan) and the corresponding single-color images (grey). Yellow line demarcates GL7 staining and magenta line IC-PE localization (n = 4 mice). Lower row shows the merged image of anti-PD1 (yellow), IC-PE (magenta) and anti-CD21/35 (cyan) and the corresponding single-color images (grey). Yellow line demarcates PD1 staining and magenta line IC-PE localisation (n=4 mice). C) FDC numbers in non-transgenic C57BL/6 (WT; n = 5) and BCR-transgenic B1-8f (B1-8^flox^Igκ^-/-^; n = 6) and MD4 mice (n = 2) 24 hours after immunization with IC-PE. D) Representative confocal images of LNs from non-tg (WT), B1-8f and MD4 mice 24 hours after immunization with IC-PE (magenta). FDC networks are shown in cyan (anti-CD21/35). The white line delimits the edges of the organs. E) Percentage of GC B cells in non-immunized (light grey; n = 4 mice) and IC-immunized mice treated with anti-CD4OL (orange; n = 8 mice) or isotype control antibody (black; n=8 mice) as described in Figure 2B. Quantitative data show means ± SD and analysis by two-tailed one-way ANOVA with multiple comparisons.

**Figure 2:**
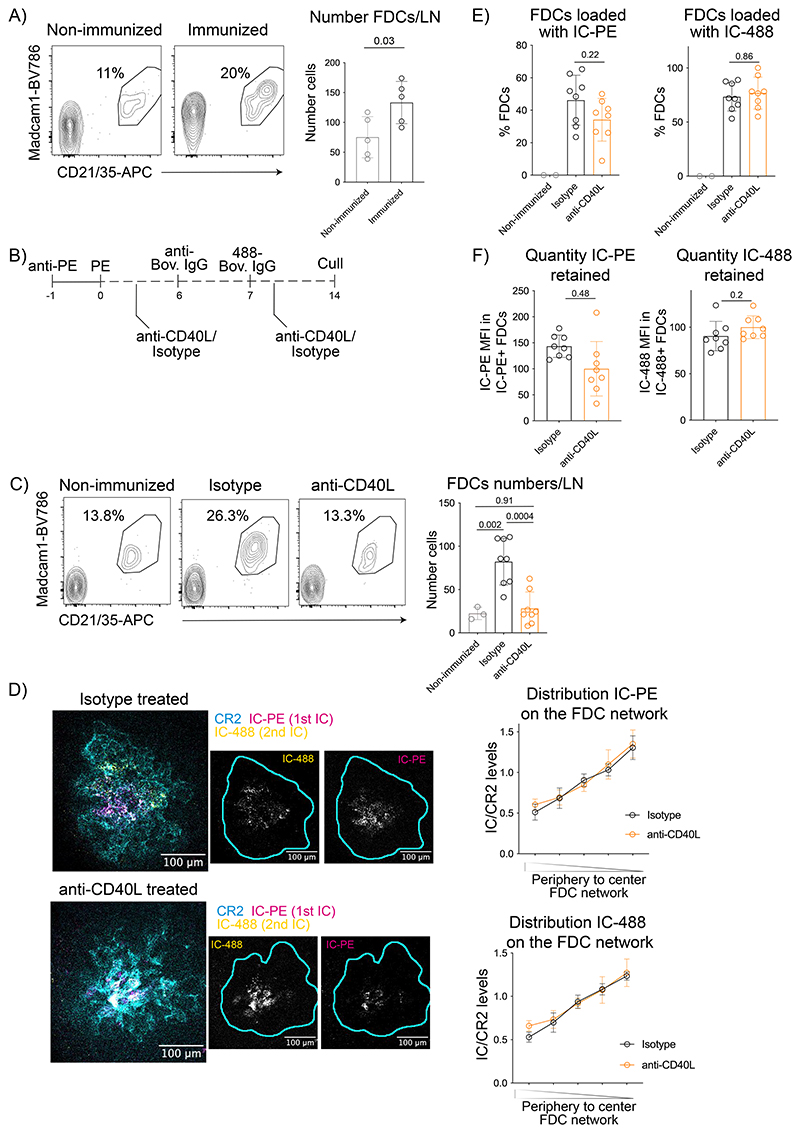
CD40L signals induce FDC network expansion and activation but do not regulate antigen centralization or retention. A) Flow cytometry plots gated on FDCs as shown in Extended Data Figure 1H and numbers of FDCs in LNs of non-immunized mice and mice immunized with IC-PE 13 days post-immunization (n = 5 mice). B) Experimental workflow to analyze the effect of CD40 signalling on antigen retention by FDCs. Mice were immunized first with IC-PE and then treated with anti-CD40L or isotype control antibodies (200 μg/mouse; 2 subsequent days). Seven days after the first immunization, mice were injected with the second IC (IC-Bovine-488) and treated again with anti-CD40L or isotype control. Draining LNs were analyzed 7 days after the second immunization. C) Flow cytometry plots and numbers of FDCs in non-immunized mice (grey; n = 3 mice) or mice immunized and treated with either anti-CD40L antibody (orange; n = 8 mice) or isotype control (black; n = 8 mice) as described in (B) (2 experiments). D) Confocal images and quantification of the ICs distribution in mice treated as described in (B). Single-color images (grey) show the localization of IC-PE and IC-488. Cyan line demarcates the FDC network boundary. Right graph shows the distribution of the first antigen (14 days post injection), left graph shows the distribution of the second (7 days post injection) (n = 8 LNs from 4 mice). E) Quantification of the percentage of FDCs loaded with IC-PE (1^st^ IC, left graph) and IC-488 (2^nd^ IC, right graph) in non-immunized (n=3) or immunized and anti-CD40L (n=8) or isotype control-treated (n=8) mice as described in (B). F) Quantity of ICs retained by FDCs measured as MFI in mice treated with anti-CD40L or isotype control antibodies (n = 8 mice). All quantitative data show means ± SD and analyses by two-tailed t-test or one-way ANOVA with multiple comparisons.

Changes in antigen localization may be driven by the reorganization of the FDC network after immunization. In agreement with previous data^10^, we observed that IC immunization increased the number of FDCs and the volume of FDC networks (Figure 2A, Extended Data Figure 2A). GC B cells (GL7^+^ Bcl6^+^) and T Follicular Helper cells (PD1^+^ Bcl6^+^) co-localized with both the central and peripheral FDCs, although some peripheral FDCs extended beyond the GC (Extended Data Figure 2B).

To understand if B cell activation promotes changes in the FDC network after immunization, we analyzed mice expressing transgenic BCRs specific for non-cognate antigens, B1-8^fl^ (B1-8^fl^ Igκ^-/-^, BCR specific for NP hapten) or MD4 (BCR specific for hen egg lysozyme) (see Methods). Both BCR transgenic mice had very few FDCs that formed poorly organized networks and were deficient in antigen capture at 24h post-immunization with IC (Extended Data Figure 2C and D). Thus, the basal level of BCR-driven B cell activation is necessary to promote the development of a well-organized FDC network, preventing a more detailed analysis of antigen dynamics.

To test the role of T cell-dependent B cell activation and GC formation, we blocked CD40 signaling by anti-CD40L blocking antibody injected after immunization^32^ (Figure 2B). Compared to isotype control, the anti-CD40L antibody completely blocked the GC reaction (Extended Data Figure 2E) and the expansion of FDCs induced upon immunization (Figure 2C). However, the anti-CD40L antibody did not affect antigen distribution on day 14 (Figure 2D). In addition, the anti-CD40L treated mice had a similar percentage of FDCs loaded with either the first or the second IC compared to the control group (Figure 2E), and their FDCs retained similar amounts of the second antigen 7 days post-injection, while containing only slightly less of the first IC 14 days post-injection(Figure 2F). Thus, CD40L-dependent signals promote FDC expansion after immunization, but are not involved in antigen centralization or retention for up to 7 days, although they may contribute at later timepoints.

### Heterogeneity in LZ FDCs orchestrates antigen retention

**Extended Data Figure 3:**
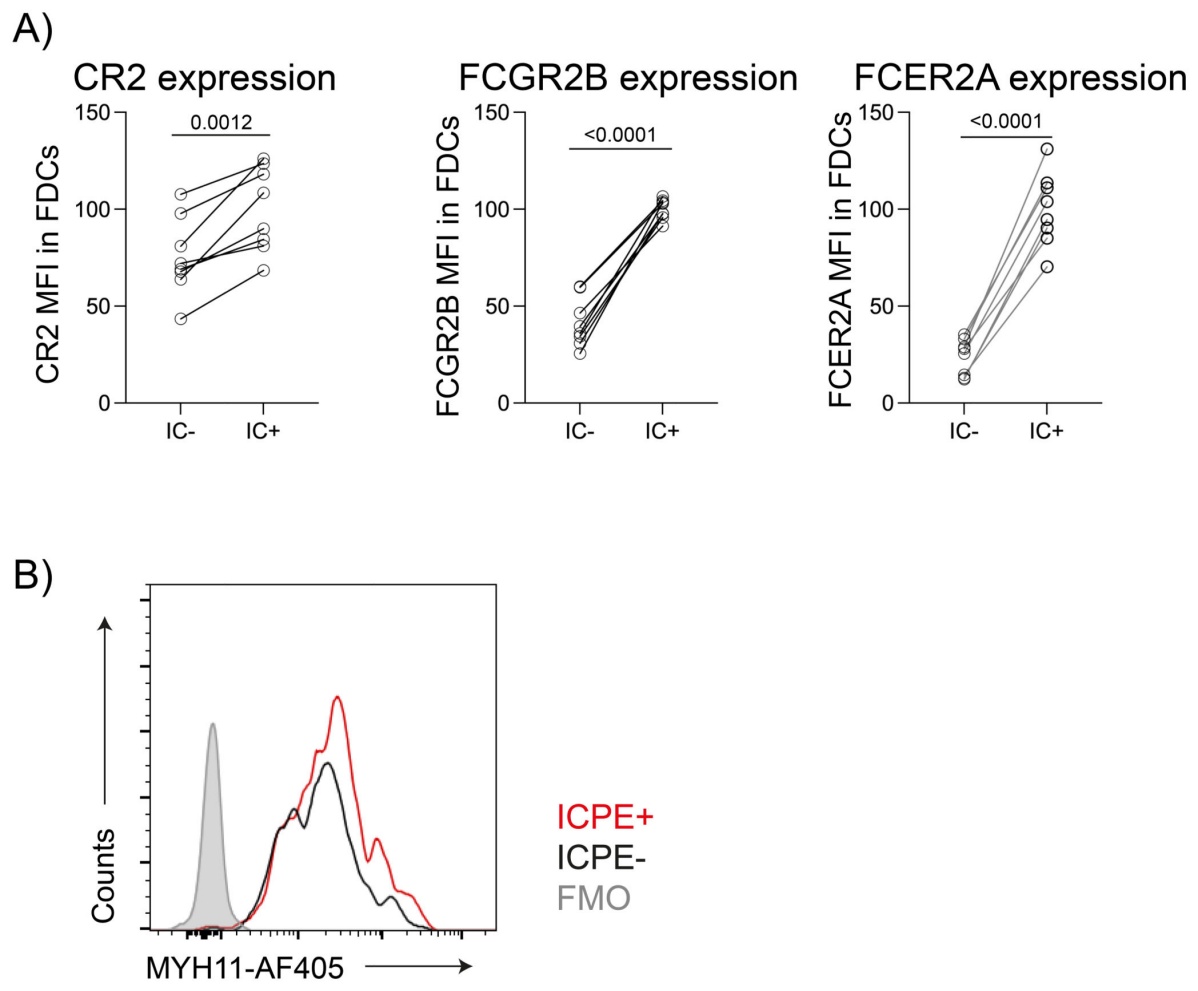
IC-binding receptor expression on FDCs. A) CR2, FCGR2B and FCER2A membrane expression on IC^+^ and IC^ FDCs 7 days after immunization (n = 7 mice; 2 experiments). B) Histogram showing Myosin heavy chain 11 (MYH11) expression in IC-PE^+^ and IC-PE^-^FDCs 7 days after immunization. Quantitative data show means ± SD and analysis by two-tailed paired t-test.

**Figure 3:**
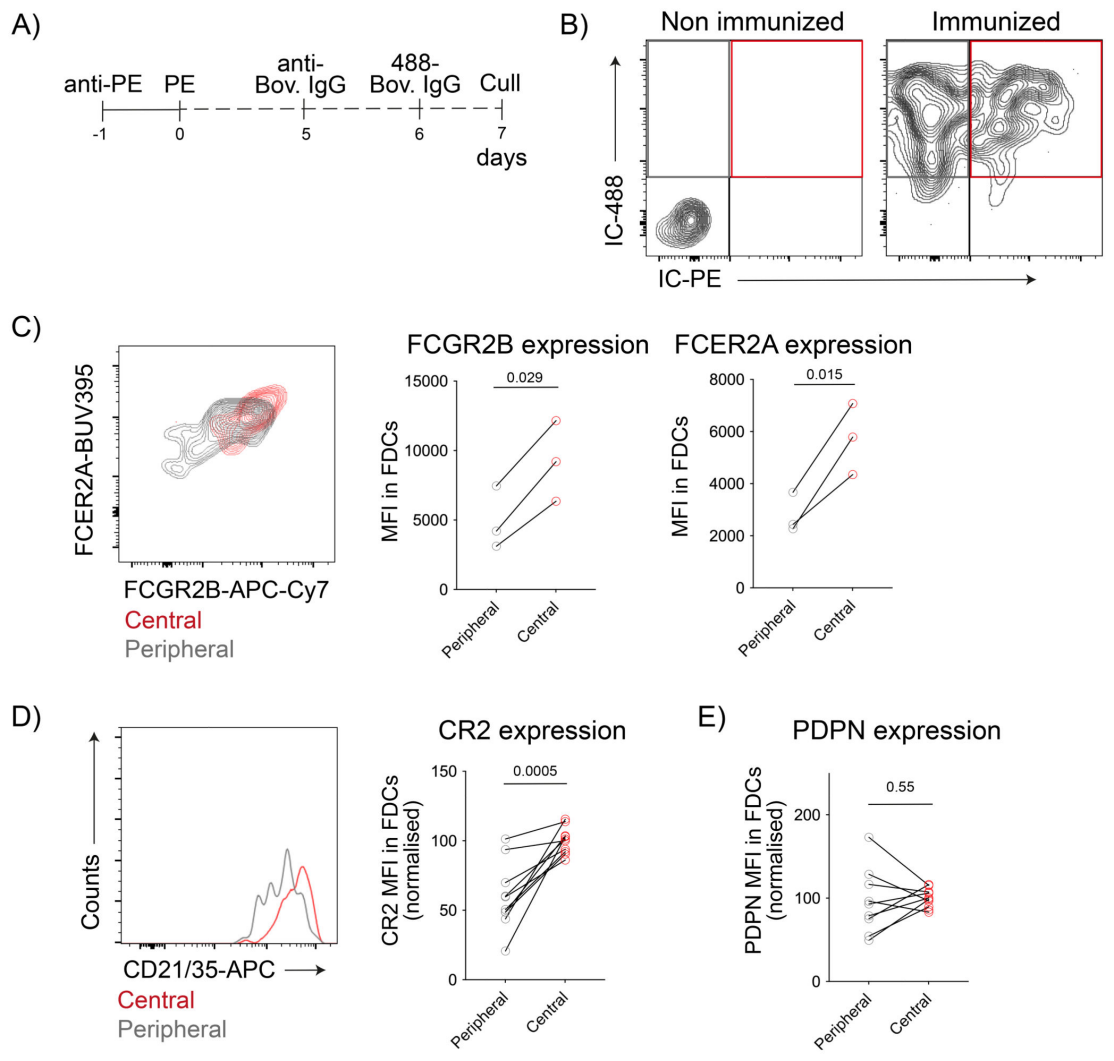
Central and peripheral LZ FDCs express different levels of IC-binding receptors. A) Immunization workflow to distinguish peripheral and central FDCs by flow cytometry. Mice were immunized first with IC-PE, followed by IC-488 6 days later. LN were analyzed 24 h after the second immunization. B) Flow cytometry gating strategy to identify peripheral and central FDCs in LNs of immunized mice following workflow described in (A). Retention of the first IC (IC-PE) distinguishes central (IC-PE^+^ IC-488^+^, red gate) from peripheral FDCs (IC-PE^-^ IC-488^+^, grey gate). C) FCGR2B (stained with anti-CD16/32 antibodies) and FCER2A (stained with anti-CD23 antibodies) membrane expression on central (red) and peripheral (grey) FDCs from mice immunized as described in (A) and (B) (n = 3 mice). D) CR2 membrane expression on FDCs as described in (A) and (B) (n = 10 mice, 3 experiments). E) PDPN membrane expression on FDCs as described in (A) and (B) (n = 10 mice, 3 experiments). P values are from a two-tailed paired t-test.

To explore the differences between the peripheral and central FDCs, we took advantage of sequential immunizations on days 0 and 6 with two differently labelled antigens (Figure 3A). One day after the last immunization, central FDCs contain both antigens while peripheral FDCs contain only the last, allowing us to distinguish both populations by flow cytometry (Figure 3B). Central FDCs expressed higher levels of FCGR2B, FCER2A (Figure 3C), and CR2 than peripheral FDCs (Figure 3D) but had similar levels of Podoplanin (Figure 3E). Similar observations were made when comparing FDCs that retained antigen at day 7 to those that did not (Extended Data Figure 3A).

Recently, single-cell transcriptomics revealed two subsets of FDCs: the LZ FDCs located in a peripheral area closer to the subcapsular sinus and DZ FDCs closer to the T cell zone^6,21^. LZ FDCs can be distinguished by high expression of CR2, and myosin heavy chain 11 (MYH11), whereas DZ FDCs express CXCL12 and PDLIM3 ^6,21^. To understand how the central and peripheral FDCs relate to LZ and DZ FDC subsets, we analyzed the expression of MYH11^6^. Both central and peripheral FDCs expressed high levels of MYH11, indicating that both populations belong to LZ FDCs (Extended Data Figure 3B).

**Extended Data Figure 4:**
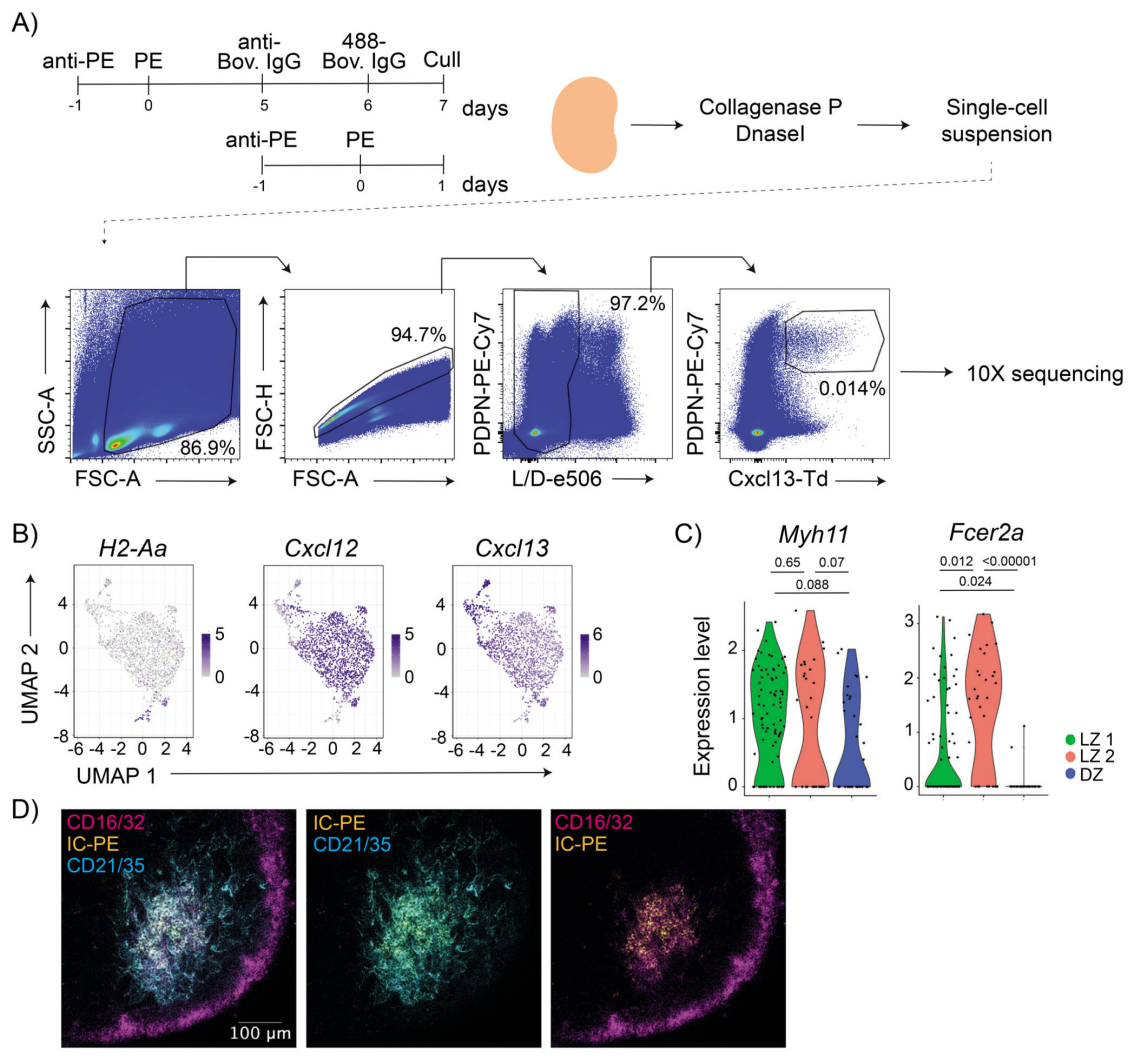
ScRNAseq of follicular stromal cells. A) Experimental workflow for scRNAseq of Cxcl13-TdTomato^+^ LN cells. Cxcl13-TdTomato mice were immunized consecutively with two ICs separated by 7 days or only with one IC. 24 h after the last immunization, draining LNs were dissociated into a single-cell suspension, stained and live cells were flow-sorted based on PDPN and TdTomato positivity. Single-sorted cells were used for 10x RNA sequencing. B) Feature plots showing expression of markers for hematopoietic cells *(H2-Aa)* and cytokines important for LN organization, *Cxcll2* and *Cxcll3*. C) Violin plots showing the expression of *Myh11* and *Fcer2a* on the three FDC clusters from Figure 4C (LZ 1 in green, LZ 2 in red, and DZ in blue). One-tail adjusted P for multiple comparisons. D) Confocal image of a LN from a mouse after 7 days postimmunization with IC-PE (yellow). CR2 staining is shown in cyan and CD16/32 in magenta (n = 4 LNs; 2 experiments).

**Figure 4:**
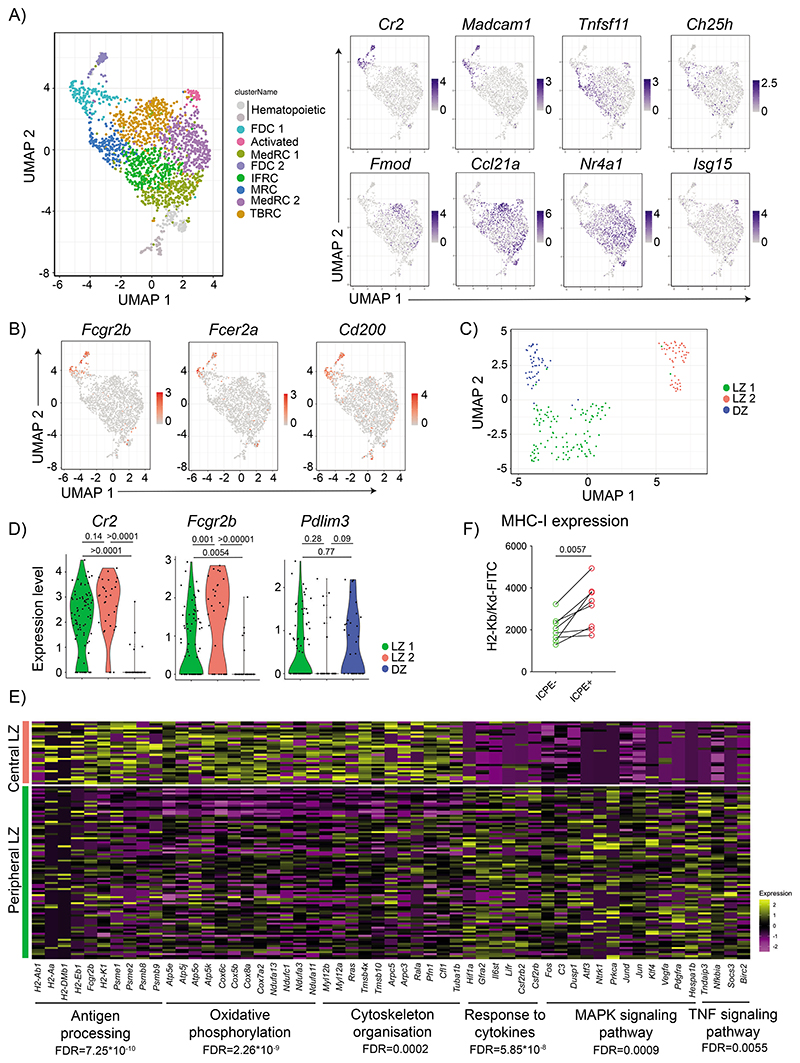
Single-cell transcriptomics differentiates two LZ FDC clusters with different functional activity. A) UMAP plot of Cxcl13-TdTomato expressing LN stromal cells. Left panel shows the merged data from mice immunized as described in Extended Data Figure 4A. Right panels show feature plots illustrating the expression of subset-specific marker genes for the indicated LN stomal cell clusters (data pooled from 3 experiments, 6-7 mice/ experiment). B) Feature plots showing the expression pattern of Fcgr2b, Fcer2a and Cd200. C) UMAP of FDC1 and FDC2 cells from (A) after subclustering. D) Violin plots showing the expression of marker genes specific for the three FDC clusters (LZ 1 in green, LZ 2 in red and DZ in blue). One-tail adjusted P for multiple comparisons. E) Heatmap of scaled expression of genes differentially expressed (>2-fold increased expression; adjusted P < 0.05) between LZ 1 and LZ 2 FDC subsets grouped into functional and signalling pathways identified by STRING (One-tail False Discovery Rate for multiple comparisons <0.01). F) MHC-I surface levels on IC-PE^+^ FDCs (central LZ FDCs) or IC-PE^-^ (peripheral FDCs) 7 days after immunization (n = 9 mice; 2 experiments). Two-tailed paired t-test.

To understand better the heterogeneity within the FDCs, we performed scRNAseq of the B follicle reticular cells marked by CXCL13-Cre TdTomato^6^. Mice were analyzed at two different time-points after immunization with antigen-IC (Extended Data Figure 4A), however, we performed all analyses on pooled samples, as we did not observe differences in gene expression between time points. Unsupervised clustering revealed 10 distinct clusters of cells. Two corresponded to contaminating hematopoietic cells identified by their lack of *Cxcl13* expression and high expression of *H2-Aa* (Extended Data Figure 4B). The other 8 clusters corresponded to follicular stromal cells of which 7 were similar to clusters previously described using similar approaches^6,7^ (Figure 4A). Assignment of these 7 clusters using subset-specific genes identified two clusters of FDCs sharing *Cr2* expression; MRCs expressing *Madcam1* and *Tnfsf11*; IFRCs showing *Tnfsf11* and *Ch25h* expression; TBRCs with the expression of *Fmod* and *Ccl21a*; and two clusters of MedRCs sharing *Nr4a1* expression. The eighth cluster corresponded to follicular stromal cells with a prominent interferon-related signature, which could correspond to an activated stromal subset.

Further analysis of the two FDC clusters indicated heterogeneity within the FDC 1 cluster, with only a fraction of cells expressing *Fcgr2b*, *Fcer2a* and *Cd200*. In contrast, these genes were expressed homogenously in FDC cluster 2 (Figure 4B). Furthermore, the cells in FDC cluster 1 that did not express these genes expressed higher levels of *Cxcl12* (Extended Data Figure 4B). Consequently, we decided to re-embed FDC 1 and 2 cells and perform subclustering, which grouped the cells into three FDC clusters (Figure 4C). We assigned one cluster to DZ FDCs based on the low *Cr2* and high *Pdlim3* expression, and two clusters to LZ FDCs based on their high *Cr2* and *Myh11* expression (LZ 1 and LZ 2) (Figure 4D and Extended Data Figure 4C). Even though the differences in *Pdlim3* and *Myh11* expression between clusters were not significant because of the low number of cells, the combination of *Pdlim3*, *Myh11,* and *Cr2* allowed us to discriminate the three populations. LZ 1 cluster expressed lower levels of *Fcgr2b* and *Fcer2a*, relating this population to peripheral FDCs, while LZ 2 cluster expressed higher levels of *Fcgr2b* and *Fcer2a*, relating them to the central FDC (Extended Data Figure 4C, Figure 4D, 3C). To confirm the relationship of the LZ 1 and LZ 2 clusters to peripheral and central FDCs in situ, we stained clarified LN 7 days after IC-PE immunization with antibodies specific for FCGR2B. Images showed that central FDCs that retained antigen colocalized with the brightest FCGR2B-expressing cells, indicating that the LZ 2 cluster corresponds to central FDCs (Extended Data Figure 4D).

Analysis of the pathways enriched among differentially expressed genes (DEG) using STRING^33^ (Figure 4E) showed that central LZ 2 FDCs upregulated molecules related to antigen presentation to B cells, but also to T cells, such as MHC-I and MHC-II related genes, together with several subunits of the proteasome complex. An increase in MHC-I expression on central versus peripheral FDCs was also observed at the protein level by flow cytometry (Figure 4F). Central LZ FDCs also upregulated expression of genes involved in the mitochondrial respiratory complex participating in oxidative phosphorylation and genes controlling cytoskeleton organization, which could explain their high degree of dendritic organisation and compaction. On the other hand, peripheral LZ FDCs seemed more responsive to extracellular signals with the upregulation of different cytokine receptor genes and intermediaries of MAPK and TNF signalling pathways (Figure 4E). These results suggest that two populations exist within LZ FDCs, likely corresponding to the central and peripheral FDCs identified by imaging.

### Both peripheral and central FDC show low antigen degradation

**Extended Data Figure 5:**
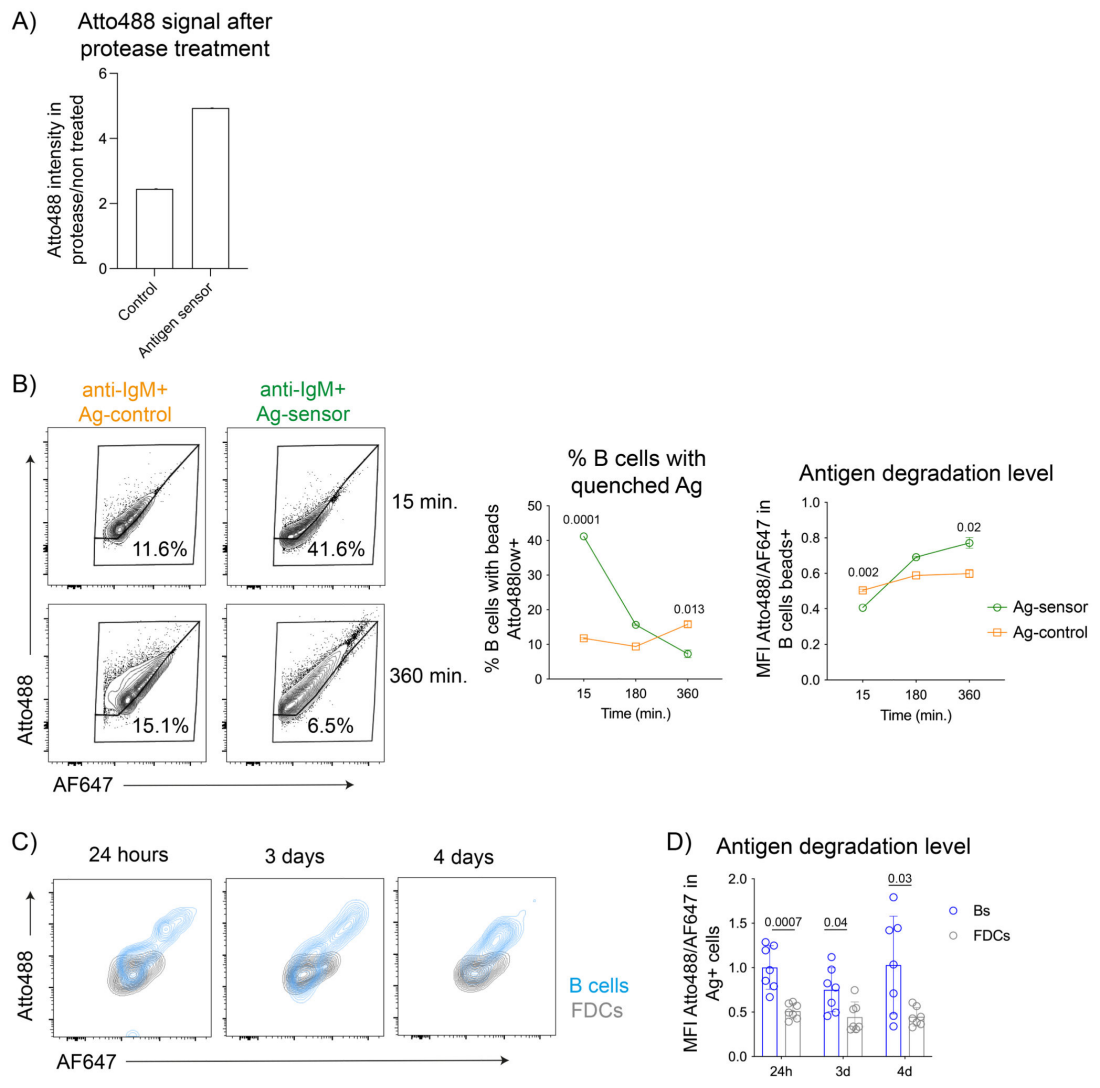
Antigen degradation by FDCs. A) Quantification of Atto488 intensity after treating the control sensor lacking the BHQ-1 quencher or antigen-degradation sensor (1:9 antigen:quencher molar ratio) with protease for 30 min at 37°C. B) Naïve B cells were incubated with beads coated with anti-IgM and the antigendegradation sensor (green) or control sensor (orange) at indicated times at 37 °C. Plots illustrate Atto488 and AF647 intensity on B cells containing degradation-sensor beads or control sensor beads. Graphs show the percentage of B cells containing quenched antigen (% of Atto488 low) and the levels of antigen degradation on B cells containing beads (measured as Atto488/AF647 intensity ratio) (2 experiments). C) Contour plots show Atto488 and AF647 levels on B cells (blue) and FDCs (grey) containing IC-antigen-degradation sensor at different time points post-immunization. D) Quantification of the antigen degradation levels in antigen^+^ B cells and FDCs at different time points post-immunization. (n = 7 mice; 2 experiments)). All quantitative data show means ± SD analyzed by two-tailed unpaired t-test.

**Figure 5:**
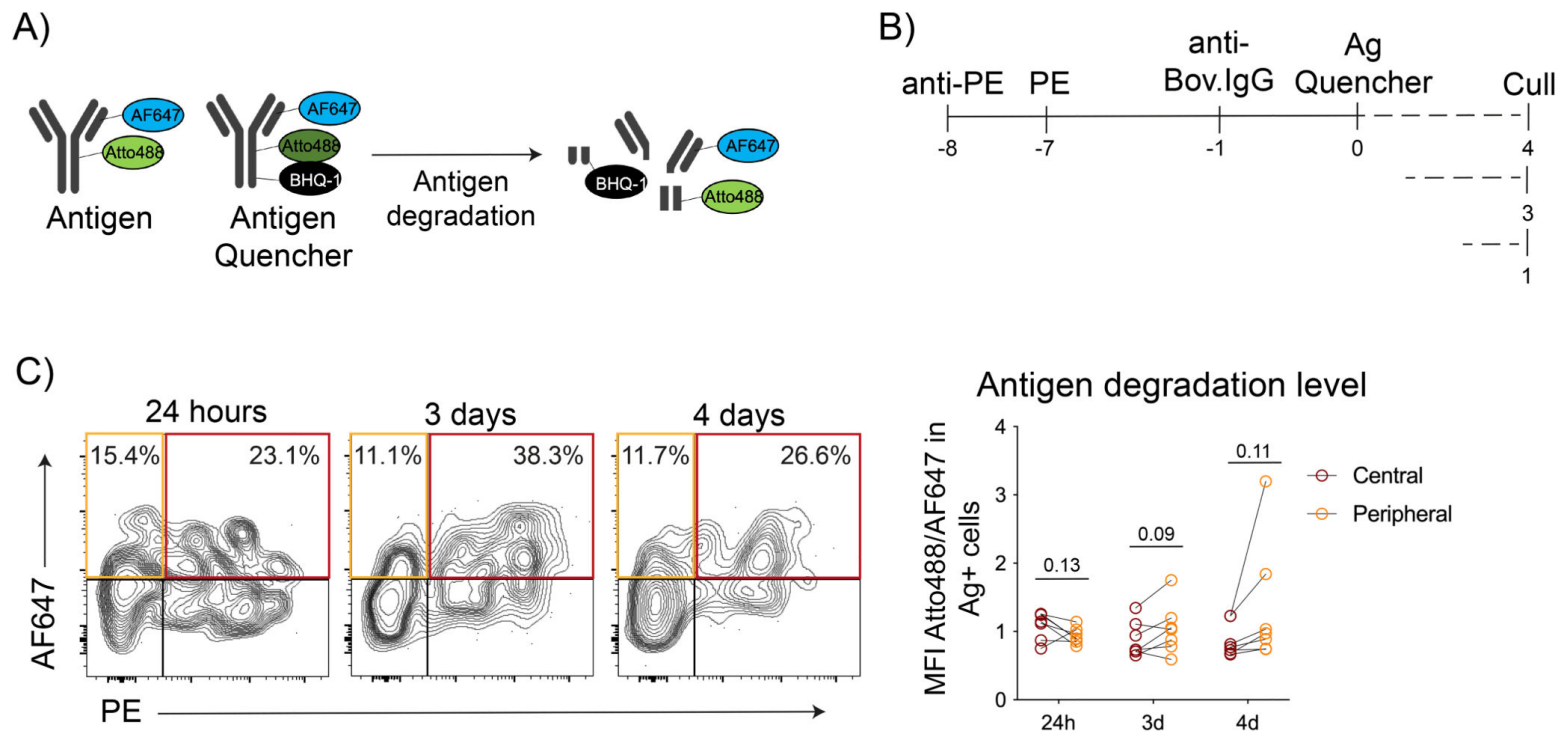
Central and peripheral LZ FDCs show a similarly low ability to degrade antigens. A) Schematics of the antigen-degradation sensor and a control sensor lacking the quencher. The antigen (bovine-IgG) is covalently bound to AF647 and Atto488 dyes. The coupled BHQ-1 quencher absorbs Atto488 emission. Upon degradation, BHQ-1 is separated from Atto488, releasing its fluorescence from quenching. B) Immunization workflow to test the level of antigen degradation by central and peripheral LZ FDCs 24-hours, 3- and 4-days after immunization with the IC-antigen-degradation sensor. C) Left, flow cytometry gating on central (red) and peripheral (orange) FDC populations. Right, the levels of antigen-sensor degradation on central (IC-PE^+^ IC-antigen sensor^+^ FDCs; in red) and peripheral (IC-PE-IC-antigen-sensor^+^ FDCs; in orange) FDCs in mice immunized as described in (B) (n = 7 mice, 2 experiments). Quantitative data show means ± SD and analysis by two-tailed paired t-test.

To understand the mechanisms underlying the selective retention of antigens by central FDCs, we compared the ability of FDC populations to keep antigens in their native conformation. We generated an antigen-degradation sensor by labeling an antigen (bovine-IgG) with Atto488 and its quencher (BHQ-1), along with a non-quenchable dye AF647 (Figure 5A). Atto488 fluorescence was efficiently quenched, but recovered after proteolysis (Extended Data Figure 5A). We conjugated the sensor to anti-IgM-coated beads, which can be phagocytosed and processed by B cells^34^. After incubation with B cells, the percentage of B cells containing quenched antigen (Atto488-low cells) decreased over time (Extended Data Figure 5B), while the total antigen degradation increased compared to beads containing a control sensor lacking the quencher. Thus, the sensor detects physiological levels of antigen degradation.

To measure antigen degradation by FDCs in vivo, mice were first immunized with IC-PE to differentiate central and peripheral FDCs and, 7 days later, immunized with the antigen-degradation sensor IC. LN cells were analyzed at 24 hours, 3- and 4-days (Figure 5B). As expected, FDCs showed lower antigen degradation compared to B cells (Extended Data Figure 5 C and D). However, IC-PE-positive central and IC-PE-negative peripheral FDCs showed similar sensor degradation (Figure 5C), indicating that the loss of antigen in the periphery of the FDC network is not caused by increased antigen degradation.

### CR2 density controls antigen retention

**Extended Data Figure 6:**
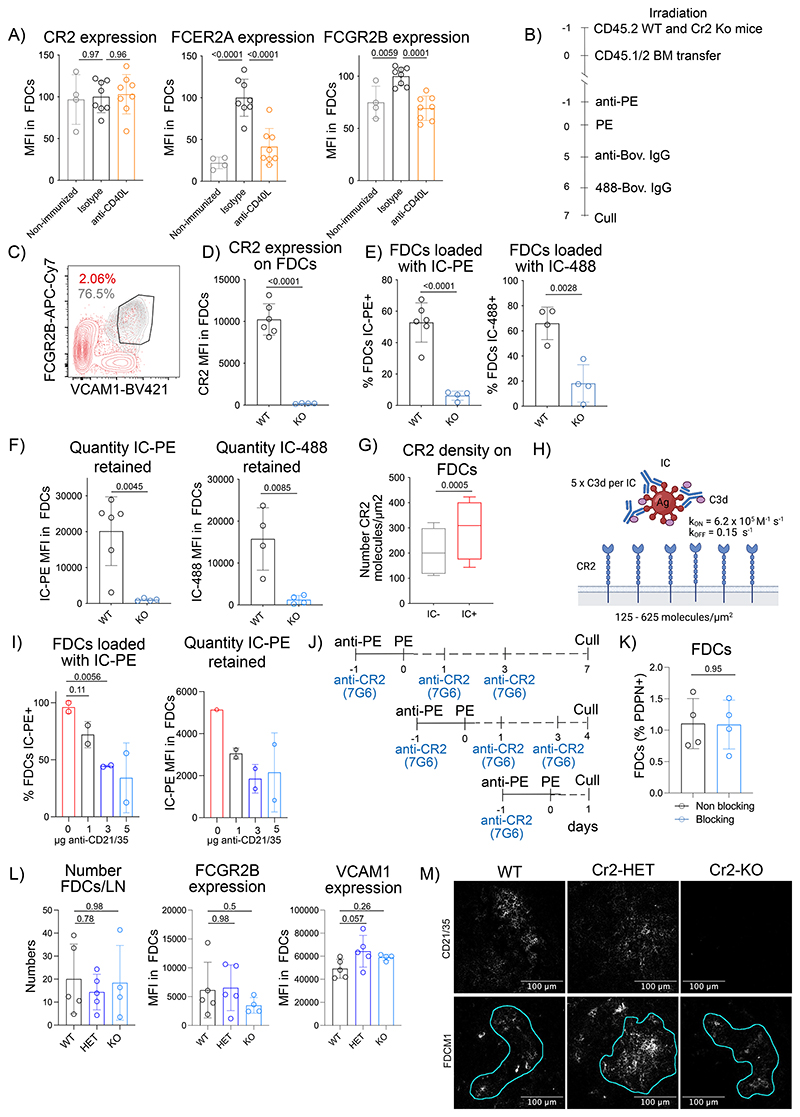
*In vivo* IC deposition on FDCs requires CR2 expression. A) CR2, FCER2A and FCGR2B membrane expression on FDCs from mice described in Figure 2C. B) Experimental workflow. Lethally irradiated CD45.2 WT(n = 6) and Cr2-KO (n = 4) mice reconstituted with bone marrow cells from WT CD45.1/CD45.2 mice and immunized with IC-PE and IC-488 6 days later. C) Gating strategy based on FCGR2B and VCAM1 expression to analyze FDCs in CD45.2 WT and Cr2-KO mice reconstituted with WT CD45.1/CD45.2 BM. In grey, FCGR2B and VCAM1 expression on WT FDCs (PDPN^+^ CD31^-^ Madcam1^+^ CD21/35^hl^); in red, on PDPN^+^ CD31^-^ stromal cells. D) CR2 expression on WT and Cr2-KO FDCs from mice as described in (B). E) Percentage of FDCs loaded with IC-PE and IC-488 from mice described in (B). F) Quantity of IC loaded in the FDC network from mice described in (B). G) Surface CR2 density on IC^+^ (red) or IC^-^ (grey) FDCs 7 days after immunization. Plots show the median (line), the 25^th^ and 75^th^ percentiles (box) and 1.5x the interquartile range (whiskers) of the number of CR2 molecules/μm^2^ (n = 8 mice; 2 experiments). H) Schematic of the binding of a C3d-coated IC to the surface of an FDC illustrating the mathematical modelling parameters. I) Percentage of FDCs loaded with IC-PE and amount of IC-PE 24h after IC-PE immunization and injection of anti-CD21/35 blocking antibody (n = 2 mice). J) Immunization workflow to analyze the effect of blocking CR2-C3d binding using an anti-CD21/35 antibody (7G6). K) Percentage of FDCs within the stromal PDPN^+^ cells in LNs from mice untreated (black) or treated (blue) for 7 days with anti-CD21/35 (7G6) blocking antibody as shown in J (n = 4 mice). L) Number of FDCs per LN and expression of FCGR2B and VCAM1 in WT (black; n = 5), Cr2-HET (dark blue; n = 5) and Cr2-KO mice (light blue; n = 4) bone-marrow reconstituted with WT CD45.1 cells. M) Representative images of a FDC network from mice described in L. Cyan line demarcates FDC network based on FDCM1 stain (n = 5 mice; 2 experiments). Quantitative data show means ± SD and analysis by two-tailed unpaired t-test or Oneway ANOVA with multiple comparisons.

Central FDCs may retain antigens better due to the lower dissociation of ICs from their surfaces. This could involve the enhanced expression of FCGR2B on central FDCs after immunization. However, enhanced expression of FCGR2B required CD40L-induced signaling (Extended Data Figure 6A), whereas ICs retention did not (Figure 2D-F), making a contribution from FCGR2B unlikely.

**Figure 6:**
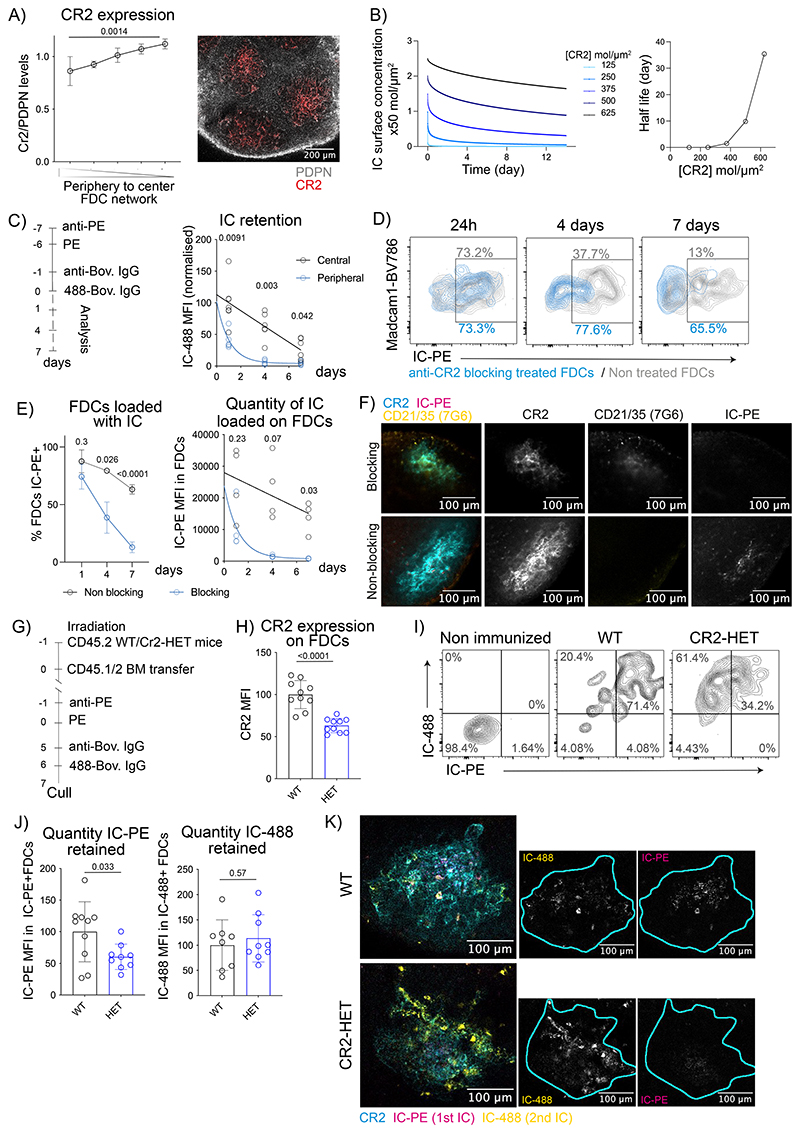
Membrane levels of CR2 dictate the half-life of antigen retention on peripheral and central FDCs. A) Quantification of CR2 levels on the FDC network in non-immunized mice (n = 4 mice). The confocal image shows PDPN (grey) and CR2 (red). B) Modeling IC dissociation from FDC surfaces. Left, surface concentrations of ICs retained on FDCs over time depending on CR2 levels. Right, IC half-life versus CR2 surface concentration (molecules/μm^2^). C) Rates of IC loss from central and peripheral LZ FDCs. Left, immunization workflow. Graphs show the quantity of the second IC retained by central FDCs (IC-PE^+^ IC-488^+^, in grey) and peripheral FDCs (IC-PE^-^ IC-488^+^, in blue) (n = 7 mice; 2 experiments). Lines show non-linear regression fits to the data. D) Flow cytometry plots show IC-PE levels on gated FDCs from mice immunized with IC-PE and treated or not with anti-CD21/35 blocking antibody. E) Quantification of the percentage of IC-PE^+^ FDCs and quantity of IC-PE on the total FDCs (n = 4 mice) from mice treated as D. F) Representative images (n = 4 mice) of FDC networks from LNs of mice treated as in D. Anti-CD21/35 staining (7E9, cyan), IC-PE (magenta),injected anti-CD21/35 (7G7) antibody (yellow). Single-color images are shown in grey. G) Experimental workflow for measuring antigen retention by Cr2^+/+^ (WT) and Cr2^+/-^ (HET) FDCs. Bone-marrow reconstituted lethally irradiated CD45.2 WT and Cr2-HET mice were immunized with IC-PE and 7 days later with IC-488. H) CR2 membrane expression on FDCs from WT (black) and Cr2-HET (blue) mice as described in (G) (n = 10 mice). I) Gating strategy to analyze the IC loading in non-immunized WT mice, and WT or Cr2-HET mice immunized as in (G). J) Quantity of IC loaded on total FDCs from WT (n=10) and Cr2-HET (n=9) mice following the experimental workflow described in (G). K) Representative images of FDC networks from mice treated as described in G. CR2 (cyan), IC-PE (magenta), IC-488 (yellow). Single-color images are shown in grey. Cyan line demarcates the FDC network boundary. All quantitative data show means ± SD and analysis by two-tailed unpaired t-test and two-way ANOVA.

In contrast, localized antigen retention could involve enhanced surface levels of CR2 on central FDCs (Figure 3D, Extended Data Figure 3A), which were independent of immunization or CD40L blockade (Extended Data Figure 6A). We confirmed that central FDCs express more CR2 than peripheral FDCs at the steady-state by analyzing CR2 expression in clarified LNs from non-immunized mice by microscopy and normalizing to PDPN (Figure 6A), which was similarly expressed on all FDCs (Figure 3E).

To validate that the retention of antigen-ICs by FDCs was CR2-mediated, lethally irradiated CD45.2^+^ WT and *Cr2* knock-out (KO) mice were bone-marrow reconstituted with CD45.1/2 WT haematopoietic cells and immunized with 2 subsequent antigen-ICs (Extended Data Figure 6B). Since stromal cells are radioresistant, bone marrow transplantation restricts the *Cr2*-deficiency to the FDC compartment^35,36^. To analyze antigen binding to *Cr2*-deficient FDCs, we identified FDCs based on the expression of FCGR2B and VCAM1 (Extended Data Figure 6C). FDCs from *Cr2*-KO mice didn’t express CR2 (Extended Data Figure 6D) and showed negligible IC binding at 24 h or 7 days post-immunization (Extended Data Figure 6E and F). Thus, the antigen-IC retention observed in this immunization model was exclusively CR2-dependent.

To understand if the ~1.5-fold difference in CR2 levels on the cell surfaces of peripheral versus central FDCs (Figure 3D, Extended Data Figure 3A) could be responsible for different rates of antigen loss from these cells over time, we generated a stochastic model to predict the probability of IC survival on FDC surface over time as a function of CR2 surface density. The model used values from quantitative flow cytometry of CR2 (Extended Data Figure 6G), CR2 dissociation (K_OFF_) and association (K_ON_) rates with C3dg, a C3 fragment closely resembling C3d, along with an estimated number of available C3d binding sites per IC (N_L_) (Extended Data Figure 6H). The model predicted that elevated CR2 levels proportionally increase the initial loading of ICs onto FDCs (Figure 6B, left) and non-linearly increase IC retention over the next 14 days. Plotting the inferred IC half-lives confirmed their sensitivity to CR2 density with a non-linear rise starting around their physiological levels on FDCs (Figure 6B, right). A 1.5-fold increase in CR2 density, from 250 to 375 molecules per μm^2^, similar to the difference between peripheral and central FDCs, led to an increase in the apparent half-life of the IC on the FDC surface from 1.2 hours to 1.5 days and in 6.7-fold higher levels of IC on day 14. Thus, even subtle differences in CR2 surface levels on FDCs can dramatically impact IC dissociation at long time scales.

To quantify the rates of IC loss from FDCs experimentally, we measured IC-488 antigen levels on peripheral and central FDCs from mice immunized first with IC-PE, then with IC-488 7 days later, and sacrificed 1, 4, or 7 days afterwards (Figure 6C). Peripheral FDCs had less antigen on day 1 and the antigen disappeared faster thereafter as compared to the central FDCs, as the model had predicted.

We used two strategies to test the role of CR2 density in antigen retention. First, we used an anti-CD21/35 blocking antibody titrated to reduce the IC binding capacity on central LZ FDCs approximately by 50%, similar to the levels observed on the peripheral LZ FDCs in untreated mice (Extended Data Figure 6I). Mice treated with blocking anti-CD21/35 antibodies were immunized with IC-PE and analyzed after 1, 4, or 7 days (Extended Data Figure 6J). The treatment didn’t affect the numbers of the FDCs (Extended Data Figure 6K, Figure 6F), however, the percentage of FDCs loaded with IC-PE, and the antigen quantity decayed faster in the anti-CD21/35 treated mice (Figure 6D-F), following kinetics similar to the peripheral FDCs in untreated mice (Figure 6C). This effect was unlikely a consequence of disturbed GCs because blocking the GC doesn’t affect antigen retention (Figure 2D-F). Second, we manipulated CR2 levels selectively on FDCs by reconstituting lethally irradiated CD45.2 WT or *Cr2*-heterozygous (HET) mice with WT CD45.1/2 bone marrow and immunizing them with two subsequent antigen-ICs as above (Figure 6G). Radioresistant FDCs from *Cr2*-HET mice expressed approximately 60% of WT CR2 levels (Figure 6H). We observed a significant reduction in IC-PE quantity displayed by *Cr2*-HET FDCs 7 days after immunization, which was not observed at early time-points (IC-488) (Figure 6 I-K). Thus, *Cr2*-HET FDCs had a similar ability to capture but an increased loss of ICs within 7 days after immunization compared to WT. To exclude that the reduction in Cr2 expression could be affecting the development and maturation of FDCs, we analyzed FDCs from WT, *Cr2*-HET and *Cr2*-KO mice reconstituted with WT bone-marrow cells. We observed normal FDC numbers, FCGR2B and VCAM1 expression, and network architecture between the three genotypes (Extended Data Figure 6L, M).

Collectively, these data indicate that the small differences in the steady-state levels of CR2 expressed by peripheral and central FDCs result in preferential retention of antigens in the center of the follicle over time because of slower dissociation of ICs from central FDCs.

### Repeated immunizations compete for CR2 on central FDCs

**Extended Data Figure 7:**
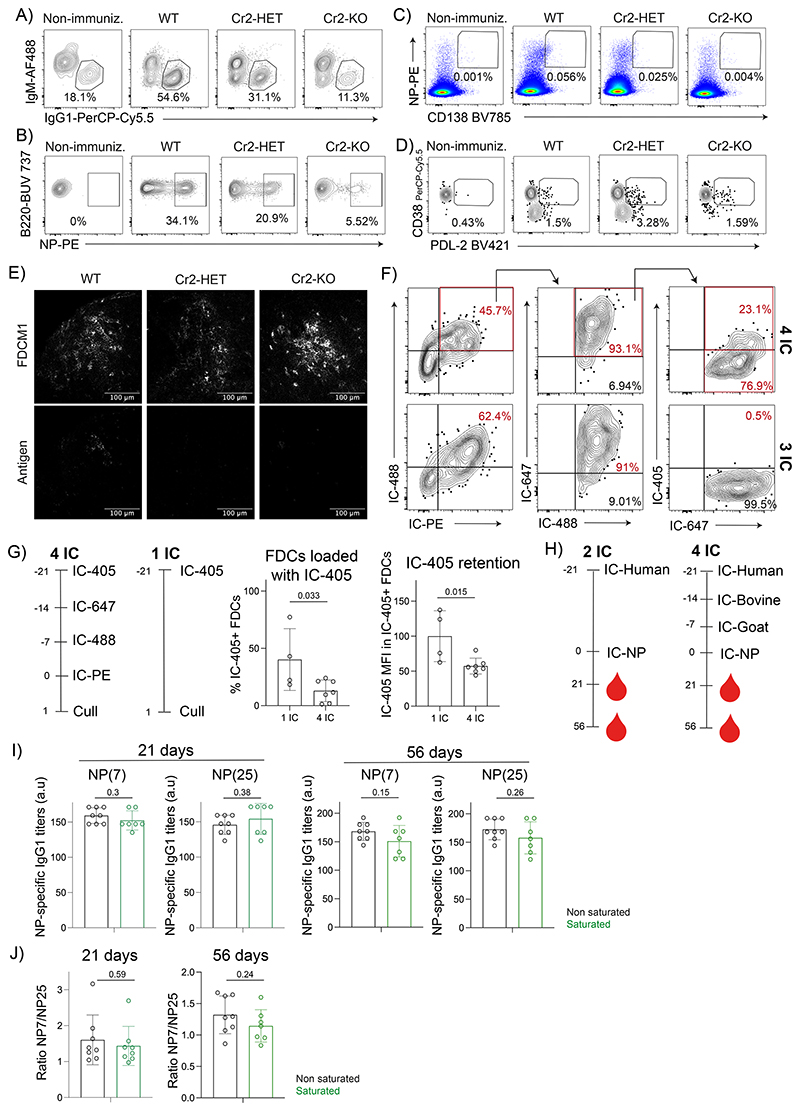
Central FDCs can get partially saturated. A) Gating strategy to analyze IgG1^+^ cells within GC CD45.1^+^ donor B cells in WT, Cr2-HET and Cr2-KO mice bone-marrow reconstituted with CD45.1^+^ WT cells and immunized for 21 days with IC-NP. Non-immunized mice shown as control. B) Gating strategy to analyze NP-specific B cells within GC CD45.1^+^ donor B cells from mice described in A. C) Gating strategy to analyze plasmablasts (CD138+ NP+within CD45.1^+/^^) from mice described in A. D) Gating strategy to analyze memory B cells (CD38^hl^ PDL-2^+^ within the NP-specific CD45.1 ^+^ donor cells) from mice described in A. E) Representative confocal images of FDC networks from LNs of WT, Cr2-HET and Cr2-KO mice bone-marrow reconstituted with WT CD45.1 cells and immunized for 21 days with IC NP-PE (n = 3 mice). Upper panel shows FDCM1 staining. Lower panel shows NP-PE. F) Gating strategy to analyze FDCs containing different combinations of ICs from consecutive immunizations with three (lower panel) or four (upper panel) different fluorescent antigen-ICs as indicated in Figure 7A, B. G) Workflow to analyze antigen displacement by subsequent immunizations. Mice were immunized with IC-405 alone or followed by four subsequent immunizations with different ICs. Graphs show the percentage of FDCs loaded with the first antigen and the quantity (MFI) of the loaded antigen in the two groups of mice (n = 7 mice). H) Immunization workflow to analyze the antigen-specific antibody response generated to NP under non-saturating (2-IC) or saturating conditions (4-IC). I) Titers of high-affinity (NP(7)-BSA) and total (NP(25)-BSA) NP-specific IgG1 in sera of mice 21 and 56 days after immunization with either two antigen-ICs (No saturation condition; black; n = 8 mice) or four antigen-ICs (Saturation condition; green; n = 7 mice) as in D. J) Ratio of binding to NP(7) and NP(25), as measured by ELISA in mice immunized as in I. Quantitative data show means ± SD and analysis by two-tailed unpaired t-test with multiple comparisons.

**Figure 7:**
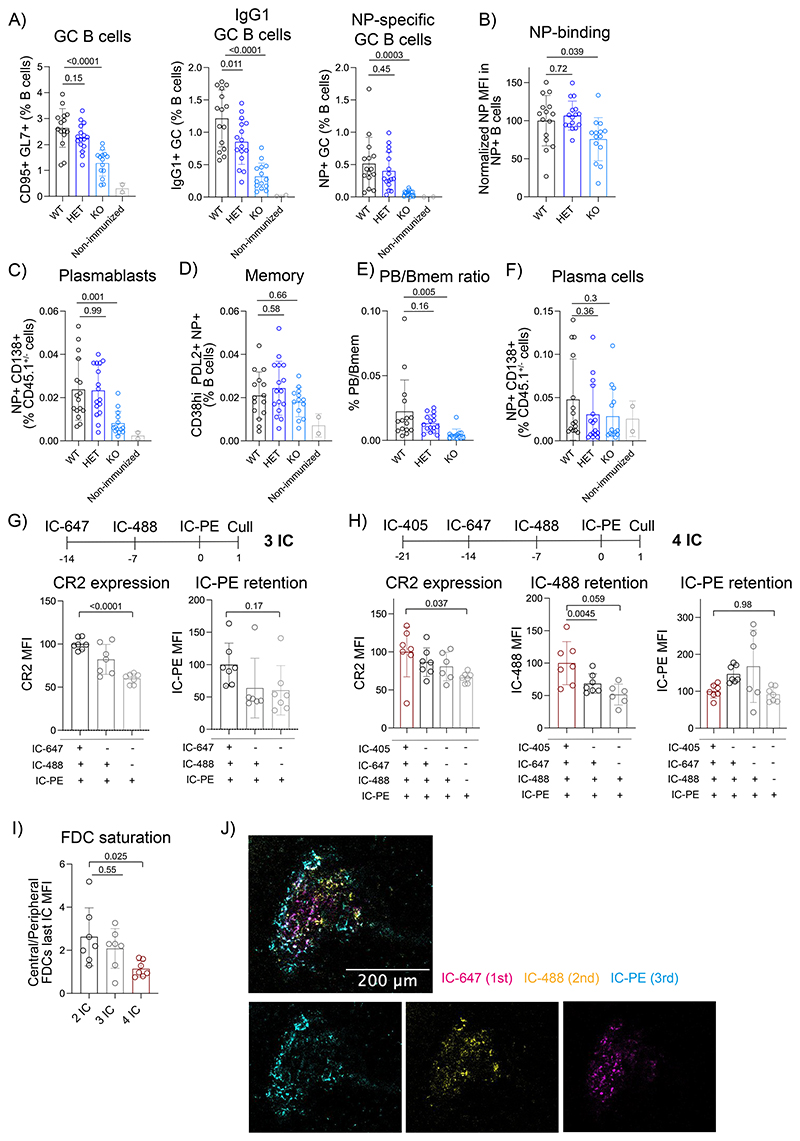
Antigen display by FDCs regulates the B cell response while successive immunizations partially saturate central FDCs. A) Percentages of GC B cells (CD95^+^ GL7^+^), IgG1^+^ GC B cells and NP-specific GC B cells in the LNs of WT (black; n=15 mice), Cr2-HET (dark blue; n = 16 mice) and Cr2-KO (light blue; n=14 mice) mice reconstituted with WT CD45.1^+^ donor bone-marrow and immunized with IC NP-PE 20 days prior to analysis. Cells were gated on CD45.1^+^ B220^+^ donor cells. B) Quantification of the NP-PE MFI in NP^+^ B cells from mice described in A. C) Percentage of plasmablasts (CD138^+^ NP^+^ within CD45.1^+/-^) from mice described in A. D) Percentage of memory B cells from mice described in A. E) Quantification of plasmablast to memory B cells ratio in WT (black), Cr2-HET (dark blue) and Cr2-KO (blue light) mice treated as described in A. F) Quantification of the bone-marrow plasma cells (NP^+^ CD138^+^ within B220^-^ TCRß^-^) from mice described in A. G) Schematic workflow of mice consecutively immunized with three distinct ICs. Plots show CR2 expression and retention of the last injected IC (IC-PE) in the different FDC subsets identified by the presence of all the three injected ICs (black), the last 2 ICs (dark grey) or only the last one (light grey) (n = 7 mice). H) Schematic workflow of mice injected consecutively with four different ICs. Plots show CR2 expression and retention of the penultimate IC (IC-488) or the last IC (IC-PE) in the FDC subsets containing four injected ICs (red), the last three ICs (black), the last two ICs (dark grey) or only the last IC (light grey). (n = 7 mice). I) Quantification of the central FDC saturation by measuring the ratio between the quantities of the last IC on central and peripheral FDCs in mice immunized with 2, 3 or 4 ICs. (n = 7 mice) J) Confocal image of an FDC network from a mouse immunized with three different fluorescent antigen-ICs as described in (G). All quantitative data show means ± SD and analysis by two-tailed one-way ANOVA with multiple comparisons.

To determine how manipulation of FDC CR2 levels affects B cell responses to antigens, we bone-marrow reconstituted WT, *Cr2*-HET, and *Cr2*-KO mice with CD45.1 WT bone-marrow cells, immunized them with NP-PE antigen-ICs and analyzed the GC response at day 20 postimmunization. We focused the analysis on CD45.1^+^ donor WT B cells, which were reconstituted similarly in all groups of mice. In mice where FDCs do not display the antigen (*Cr2*-KO), we observed a strong reduction in the percentage of GC B cells, class-switched IgG1^+^ GC B cells, and NP-specific B cells compared to WT (Figure 7A, Extended Data Figure 7A, B, E). The intensity of B cell binding to NP as a readout of BCR affinity was also reduced in *Cr2*-KO mice (Figure 7B) and so was the percentage of plasmablasts (PB) (Figure 7C, Extended Data Figure 7C). In contrast, the percentage of antigen-specific memory B cells was unaffected in *Cr2*-KO mice (Figure 7D, Extended Data Figure 7D), inducing a skewed differentiation output compared to WT mice (Figure 7E). Consistently, the percentage of antigen-specific PCs in the bone marrow of the *Cr2*-KO mice was also reduced, even though the differences didn’t reach significance (Figure 7F).

In the Cr2-HET animals, where antigens are lost faster from FDCs, there was a milder but consistent trend for reduction of the B cell response (Figure 7A-F). In particular, the class-switched GC response was significantly decreased compared to WT mice, supporting the idea that antigen persistence promotes a prolonged GC response. Even though we couldn’t detect differences in BCR affinity in the *Cr2*-HET mice (Figure 7B), there was a trend of reduced antigen-specific PB and PC differentiation contrasting with intact memory B cell differentiation (Figure 7E, F), although this did not reach statistical significance. Thus, antigen display by FDCs is essential for generating a prolonged high-affinity antigen-specific GC response that supports PC differentiation. The duration of the antigen display is also important, although less than the initial FDC antigen capture, possibly because small amounts of retained antigen are sufficient to support the B cell response.

Since central FDCs are the exclusive site of long-term antigen retention that supports the GC response, we wondered if they become saturated after repeated immunizations. We immunized mice consecutively with three (IC3; Figure 7G) or four (IC4; Figure 7H) differently labeled antigen-ICs in one-week intervals and analyzed their FDCs 24 h after the last immunization (Extended Data Figure 7F). FDCs that retained all antigens showed the highest CR2 expression (Figure 7G and H, left panels), suggesting that they correspond to the central FDCs. Indeed, imaging confirmed that all antigens older than a week were localized in the center of the follicle (Figure 7J). In the three-dose regime, the central FDCs containing all antigens (IC-647, IC-488 and IC-PE) were found to be loaded with more of the last antigen (IC-PE) than FDCs containing only the last two (IC-488 and IC-PE respectively), or only the last one (IC-PE) (Figure 7G, right panel). This result matched our previous finding that central FDCs contain more antigen than peripheral FDCs 1 day after immunization (Figure 6C). A similar phenomenon was detected in the four-dose regime, but only up to the third antigen, where the FDCs loaded with all antigens retained more IC-488 (the 3^rd^ antigen injected) than the other FDC populations (Figure 7H, middle panel). However, these central FDCs could not efficiently capture the fourth antigen (IC-PE), displaying similar amounts of IC-PE as the peripheral FDCs that contained only this last antigen (Figure 7H, right panel). Even though endogenous antibody responses against the injected xeno-antibodies could have cross-reactivity and possibly enhance IC formation in the subsequent doses, the selectivity of the saturation to the central FDCs suggests that this doesn’t confound the interpretation. To quantify the saturation of central FDCs directly, we calculated the ratio of the amount of antigen on central versus peripheral FDCs from immunizations with 2, 3, or 4 ICs (Figure 7I). This comparison confirmed that after four doses, the capacity of antigen capture by central FDCs decreased to about half compared to the peripheral FDCs.

To understand if old antigens also get displaced by new immunizations, we immunized mice with either one (1 IC) or four (4 IC) consecutive antigen-ICs (Extended Data Figure 7G) and analyzed the presence of the first antigen on FDCs at the end of the experiment. The percentage of FDCs loaded with the first antigen-IC (IC-405) and the amount of IC-405 on these FDCs was lower when the mice were immunized with the three additional doses than when they were not. This suggests that the new antigens enhanced the dissociation of the first antigen from FDCs, although they did not replace it completely.

To understand if the partial saturation and partial replacement of antigens on central FDCs modifies the B cell response to new antigens, we immunized mice with three subsequent antigen-ICs or only one antigen-IC and challenged them with IC-NP 21 days after the first immunization (Extended Data Figure 7H). We tracked the antibody response to NP in the two groups of mice over the next 56 days. We observed that the high-affinity NP-specific IgG1 class-switched responses (Extended Data Figure 7I) were similar between both groups of mice and so was the affinity maturation (Extended Data Figure 7J). Thus, the partial saturation of the FDCs by the previous antigens doesn’t impede the development of the B cell response to a new unrelated antigen.

## Discussion

We demonstrate a topological heterogeneity in the FDC network that controls antigen retention in mouse LNs via levels of CR2. Peripheral CR2-low FDCs retain antigens for the first few days post-immunization, while central CR2-high FDCs preserve antigens for weeks, creating a reservoir of prior immunizations in the GC. A similar pattern of antigen localization can be observed in the mouse spleen^37^ and in primate LNs^38^, suggesting that topological heterogeneity of FDCs is a common feature of B cell follicles. Although our data did not distinguish if central and peripheral FDCs represent distinct developmental subsets or a spatial gradient within a single population, the results indicate that spatial organization of the stroma contributes to the orchestration of B cell responses.

We show that the spatial FDC heterogeneity lies within the LZ FDC subset. Central FDCs likely relate to activated FDCs observed in the GC LZ of secondary follicles^22^. Formation of the GC may be one mechanism to establish this FDC spatial patterning, for example by enhanced production of LTα1β2 by GC B cells^39^. However, acute blockade of the GC and FDC activation using anti-CD40L antibodies did not affect CR2 levels or antigen retention. Alternatively, FDC patterning can be induced by FDC network remodelling. We show that immunization expands the FDC network, in agreement with previous data^10^. New FDCs arise from precursors near the subcapsular sinus^10,40^, potentially creating a centripetal maturation pattern. However, we could distinguish peripheral and central FDCs even before immunization, suggesting constitutive patterning, possibly induced by steady-state reactivity against endogenous antigens^41,42^. This notion is supported by the disruption of FDC organization in BCR Tg mice, which are unable to respond to most antigens. Studies using new genetic tools to map the origin and development of peripheral and central FDCs will be required to dissect these mechanisms in the future.

We corroborated the functional heterogeneity within LZ FDCs by single-cell transcriptomics. A cellular cluster resembling peripheral FDCs expressed genes regulating cytokine responses, indicating they are responsive to immune stimuli. In contrast, a cluster enriched in markers of central FDCs showed expression of genes regulating oxidative phosphorylation and the cytoskeleton. The latter could result in higher membrane stiffness, which enhances B cell affinity discrimination^43^. The central FDC cluster also expressed genes involved in the processing and presentation of antigens on MHC I and II, mirroring findings in human FDCs^44^. This suggests that central FDCs may interact with CD8 and CD4 T cells, although the relevance of this needs to be further investigated.

We show that the major factor that drives the selective retention of antigens by central FDCs is the higher expression of CR2 on their surfaces. The interactions of CR2 with C3d are transient (KD ~ 140 nM, half life ~ 6 s), mandating that long-term IC retention involves multivalent binding. Modelling indicates that the IC dissociation half-life rises sharply when CR2 exceeds ~ 250 molecules/μm^2^, typical of the levels detected on FDCs. A difference in avidity may also underlie FDC acquisition of ICs from non-cognate B cells^19^, which express only ~ 20 CR2 molecules/μm^2^. It is likely that CR2 avidity works in conjunction with the recycling of ICs into non-degradative endosomes^45,46^ and with the exclusion of extracellular proteases from the B cell follicle^47^. However, antigen degradation was similarly low between peripheral and central FDCs, suggesting that these factors do not contribute to spatial patterning. Similarly, enhanced FCGR2B expression by central FDCs did not seem to contribute to IC retention, which was exclusively CR2 dependent, consistently with previous data^48^. However, FCGR2B may play a regulatory role in the B cell response or become important upon reimmunization or in autoimmunity^25,49,50^.

Although GC reactions can develop independently of antigen binding to FDCs^28,51^, we show that FDC antigen retention promoted long-lasting GC responses with higher PB output and BCR affinity. This agrees with the reported importance of FDC-expressed CR2 for vaccines-induced antibody responses^27^ and with enhanced GC responses induced by vaccines designed to increase antigen deposition on FDCs^29–31^. In contrast, memory B cells were unaffected by the absence of FDC antigen retention. Since limiting access of activated B cells to antigen promotes their differentiation into memory B cells^52,53^, rapid antigen loss from FDCs may also favor the differentiation of memory B cells. Therefore, vaccine designs enhancing antigen persistence on FDCs could also skew the memory/PB ratio.

We show that repeated IC injections partially saturated central FDCs, although a small amount of new antigens could still be deposited by replacing old antigens. In contrast, peripheral FDCs remained fully receptive to new antigens and may assist the B cell response when central FDCs become saturated. Total FDC antigen capture indeed was more important for the GC than prolonged retention, based on the GC phenotypes of FDC *Cr2*-KO versus *Cr2*-HET mice. We propose that the FDC network handles repeated challenges using a range of CR2 levels, keeping a dynamic repository of past antigens but also remaining receptive to new ones, which contrasts with the idea of saturable FDC niches for GC development^54^.

Illuminating the mechanisms that regulate the development of central and peripheral FDCs, their CR2 levels and other functions will advance the understanding of the role of the follicular stroma in B cell responses to immunizations, infections and cancers. Engineering vaccines for high CR2 avidity may enhance FDC retention and immune protection^55,56^. Blocking CR2 binding, in contrast, may effectively displace old antigens from FDCs and terminate unwanted responses, such as in autoimmune diseases.

## Materials and Methods

### Mice

C57BL/6, *Cr2*-KO (*Cr2*^tm1Hmo^), CXCL13-TdTomato (Tg(*Cxcl13*-Cre/tdTomato)719Biat), B1-8fl (Igh^tm4Cgn^ Igκ^ctmicgn/tmicgn^), MD4 (C57BL/6^Tg(IghelMD4)4Ccg/J^) and CD45.1 (B6.SJL-*Ptprc^a^ Pepc^b^* /BoyJ) mice were used. To obtain Cr2-HET and CD45.1/CD45.2 mice, Cr2-KO and CD45.1 mice were bred with C57BL/6 mice. IgH^B1-8fl^ IgK^-/-^ (B1-8fl) mice have a rearranged VDJ gene segment inserted into the Ig heavy chain (IgH) locus and a neomycin resistance cassete replacing the exon encoding the constant region of the immunoglobulin kappa chain, which guarantees that the majority of B cells express a BCR recognising 4-hydroxy-3-nitrophenyl acetyl hapten (NP). IgHEL-MD4 (MD4) mice express HyHEL10 immunoglobulin heavy and light chains transgenes so that the majority of B cells recognise Hen Egg-white Lysozyme antigen (HEL). All experiments were approved by the Francis Crick Institute and UCL Ethical Review Panels and the UK Home Office using age- and sex-related mice.

To generate bone marrow chimaeras, recipient mice were lethally irradiated with two doses of 5Gy and reconstituted with 5x10^6^ donor bone marrow cells by intravenous injection. Reconstituted mice were fed with 0.2 mg/ml Baytril (Enrofloxacin) in their drinking water for 4 weeks post-reconstitution.

### Immunization

Mice were immunized intraperitoneally with 2 mg anti-antigen antibody in 200 μL of PBS (Rabbit anti-B-Phycoerythrin, cat. 100-4199, Rockland; donkey anti-goat IgG, cat. 705-001-003, Jackson ImmunoResearch; rabbit anti-human IgG, cat. 309-001-003, Jackson ImmunoResearch; anti-bovine IgG, cat. 301-001-003, Jackson ImmunoResearch). 18h later, mice were injected sub-cutaneously with 10 μg of fluorescent antigen (R-Phycoerythrin cat. P801, ThermoFisher; NP-Phycoerythrin cat. N-5070-1, LGC Biosearch Technologies; goat Fab2 IgG anti-horse, cat. 108-006-003, Jackson ImmunoResearch; rabbit anti-human IgG, cat. 309-001-003, Jackson ImmunoResearch; rabbit anti-bovine IgG, cat. 301-001-003, Jackson ImmunoResearch) in 100 μL mixed 1:1 with Imject Alum Adjuvant (cat. 77161 ThermoFisher) in the upper and lower flank to target the brachial, axillary and inguinal draining LNs. Mouse tissues were analyzed by flow cytometry, confocal microscopy and ELISA at different time-points after immunization.

### Cellular isolation

For FDCs preparation, draining lymph nodes were disaggregated into small pieces with 25G needles and collected in RPMI-1640 medium containing 2% FCS, 20 mM HEPES pH 7.2, 0.1 mg/ml collagenase P (Roche) and 25μg/ml DNase I (Sigma). Dissociated tissue was incubated at 37 °C for 60 min, recollecting supernatant every 15 min. After enzymatic digestion, cell suspensions were filtered using a 100 μm strainer and washed with PBS containing 0.5% FCS and 10 mM EDTA. Cell suspensions were used directly for staining with antibodies.

For B cell isolation, spleens were disaggregated using 40 μm strainer and treated with Ammonium-Cloride-Potassium Lysis buffer (AcK; made in house) for 5 minutes. Single-cell suspension was subsequently incubated with CD43 microbeads (Milteny Biotech), following manufacturer’s instructions.

### Staining

For flow cytometry staining, single-cell suspension was incubated with Fixable Viability Dye-e506 (Invitrogen) for 15 minutes in PBS. Cells were subsequently incubated with anti-CD16/32 (purified or labelled) and the appropriate antibodies for 20 minutes at 4 degrees in PBS containing 0.5% FCS and 10 mM EDTA for FDC analysis or PBS containing 2% FCS and 2mM EDTA for B cell analysis. The following antibodies were used for FDC and B cell phenotyping: B220 (RA3-6B2) Biolegend, CD45.2 (104) Biolegend, CD21/35 (7E9) Biolegend, PDPN (8.1.1) Biolegend, Madcam1 (MECA-367) BD Biosciences, CD31 (390) Biolegend, CD23 (B3B4) BD Biosciences, H2-Kb/Kd (28-8-6) Biolegend, CD95 (Jo2) BD Pharmigen, IgD (11-23c) Biolegend, T- and B-Cell activation antigen (GL7) BD Biosciences, CD45.1 (A20) Biolegend, CD138 (281-2) Biolegend, CD38 (90) Biolegend, PDL-2 (TY25) BD Horizon, IgG1 (RMG1-1) Biolegend, IgM (R6-60.2) BD Biosciences. Cells were analyzed using an LSR-Fortessa flow cytometer and analyzed using FlowJo. FDCs were gated by excluding hematopoietic cells (CD45+ PDPN-) and specifically B cells (B220+), and selecting those stromal cells (PDPN+), non-endothelial (CD31-), expressing high levels of CR2 (CD21/35hi) and Madcam1+ integrin receptor. Samples were acquired on an LSR Fortessa (BD Biosciences) using BD FACSDiva software v.8.0.1. For cell sorting experiments for scRNAseq, cells were sorted using FACS Aria Fusion and collected in PBS + 0.05% BSA. For analysing CR2 surface expression on FDCs and B cells by quantitative flow cytometry, LN dissociated cells were incubated with a surface antibody mix (anti-B220, anti-CD45.2, anti-podoplanin, anti-CD31, anti-Madcam1) containing anti-CD21/35-PE under saturation conditions. PE Phycoerythrin Fluorescence Quantitation Kit containing beads conjugated with four levels of PE was used to obtain a calibration curve from which the number of CR2 surface molecules in the cells of interest could be calculated. Surface CR2 density was calculated based on the calculated number of CR2 molecules per cell divided by the cell surface area measured using Imagestream.

For microscopy staining, LNs were incubated 4 hours with Antigenfix solution (Diapath) and washed and permeabilised in PBS containing 1% BSA, 1% normal mouse sera and 2% Triton X-100 for 24h. LNs were incubated with the antibody mix in the permeabilization buffer for 3 days at 22°C while shaking. Organs were subsequently washed in permeabilization buffer for 24h and incubated with RapiClear solution (1.47 RIN) for 24h at RT. Clarified organs were imaged mounted in RapiClear solution using Leica SP5 Upright or Leica SP8 Falcon Inverted microscopes. For PDPN staining, LNs were permeabilised in 4% SDS in 200mM boric acid at 37°C for 4h and labelled in 4% SDS solution for 4 days at 22°C as previously described in^57^.

For microscopy of sections, LNs were embedded in OCT and frozen at -80 °C for at least 24 h. 20 μm sections were obtained using the Cryotome (Leica) and fixed in 4% paraformaldehyde for 15 min. Sections were blocked using 2% BSA + 5% FBS in PBS (blocking buffer) for 1 h at 22°C and incubated for 2 h with the following antibodies: anti-PD1 (29F.1A12) and anti-CD21/35 (7E9) from Biolegend, and GL7 and anti-Bcl6 (K112-91) from BD Biosciences,. The FDCM1 staining was performed in three subsequent steps: tissues were first stained with anti-FDCM1 (BD) in blocking buffer, after washing, sections were incubated with anti-rat-AF488 (Cell Signalling; 1/400) for 1 h in blocking buffer, and finally were incubated with anti-CD21/35 antibody (Biolegend).

### Droplet-based single-cell RNA sequencing analysis

Sorted CXCL13-TdTomato^+^ PDPN^+^ live cells were run using the 10x Chromium (10x Genomics) system, and cDNA libraries were generated according to the manufacturer’s recommendations (Chromium Single-Cell 3’ Reagent Kit (v3.1 Chemistry)). Libraries were sequenced via Hiseq 4000 for Illumina sequencing. Raw sequencing data were processed using the CellRanger pipeline version 3 (10x Genomics) with the Ensembl GRCm38 release 89 reference transcriptome. Count tables were loaded into R and further processed using the Seurat R package version 3.1.5^58^. Samples were pooled from three independent experiments (Cxcl13-Td immunized for 24h, LNs from six mice pooled; Cxcl13-Td immunized for 7 days with a first antigen-ICs and 24h with a second antigen-ICs, LNs from six mice pooled). Subsequently, cells containing fewer than 200 distinct genes and cells with more than 10% of unique molecular identifiers stemming from mitochondrial genes were excluded. Furthermore, cells that featured at least one read count for *Lyve1, Hba-a1, Hba-a2, Krt18, Trac, Cd3d, Cldn5, Ly6c1, Egfl7, Ptprc, S100b, Cd79a, Cd79b* genes were removed to eliminate contaminating hematopoietic cells, erythrocytes, endothelial and epithelial cells as previously described^6^. After quality control and removal of contaminants, the remaining cells were retained for further processing using the default method from the Seurat package (version 3.1.5). Clusters were characterised based on described markers^6,7^. Differentially expressed genes between three FDC clusters were performed using Seurat within the samples from Cxcl13-Td mice immunized for 7 days. Pathways enriched among their differentially expressed genes (>2-fold increased expression; adjusted P < 0.05) were analyzed using STRING^33^.

### Image analysis

To study antigen distribution within the FDC network, acquired datasets were analyzed using MATLAB with ImageJ plugin. Images were subsampled in the x and y axis (4x) and a filtered to generate a mask of the FDC networks based on CD21 staining. Individual follicles were detected, labelled, and segmented into six concentric shells with the most peripheral serving as background. Antigen fluorescence was measured in the five inner rings, background subtracted, mean-normalized and then normalized by the anti-CD21 staining processed in the same manner.

To analyze FDC network volume, images from clarified organs were analyzed using Imaris where FDC networks were detected based on thresholded CD21 staining.

### Protein labelling with dyes

Antibodies were conjugated to one of several fluorophores AlexaFluor 405, AlexaFluor 488, AlexaFluor 647 NHS esters (Thermo Fisher) in Sodium Carbonate buffer, according to the manufacturer’s instructions. Excess dye was removed using Zeba 7K MWCO desalting columns (Pierce, Thermo Fisher).

For the degradation sensor, antigen was conjugated first with AlexaFluor 647 and AlexaFluor 488 NHS esters (Thermo Fisher) and with BHQ-1 quencher as previously described in^59^.

### In vitro antigen degradation assay

Conjugated Bovine IgG with AlexaFluor 647 and AlexaFluor 488 NHS esters (control) and Bovine IgG conjugated with AlexaFluor 647 and AlexaFluor 488 NHS esters and BHQ-1 NHS ester (ratio 1 IgG: 9 BHQ-1 molecules) were treated at 50°C for 30 minutes and 95°C for 5 minutes with 2mg/mL Proteinase K. Fluorescent emission was measured using The Spark multimode plate reader (Tecan).

### Ex vivo degradation assay

50 × 10^6^ carboxylated latex beads 1 μm in diameter were incubated overnight with a concentration of 20 μg/ml of anti-IgM plus 20 μg/ml antigen-quencher or only anti-IgM in 1 ml of PBS at 4°C.

Naïve purified B cells were resuspended in complete RPMI (10% FBS, 100μM non-essential amino acids (ThermoFisher), 2mM L-Glutamine (ThermoFisher), 50μM 2-Mercaptoethanol (ThermoFisher) and Penicillin-Streptomycin (GE Healthcare Life Sciences)) and plated in 96-well V-bottom plates at a concentration of 0.5 × 10^6^ cells in 50 μl. Antibody-coated beads were added to reach a bead:cell ratio of 3:1. The cellular and bead suspension were briefly centrifuged at 400 *g* and were incubated at 37°C for different time points. Subsequently, cells were washed and stained on ice^34^.

### HIV-multimeric nanoparticles

60-mer SpyCatcher-SpyTag particles were generated as described in^60^. Briefly, monomeric SpyCatcher-mi3 (kindly donated by Mark Howarth) was incubated with 3 times molar excess YU-gp120-SpyTag HIV envelope protein for 18h at 25°C in PBS and dialysed using 300 KDa MWCO membrane (Spectra/Por Float-A-Lyzer G2) in Sodium Carbonate buffer following manufacturer’s instructions. VLPs were subsequently incubated with AF-555 NHS ester for 1h at 25°C and dialysed using 300KDa MWCO membrane in PBS for 2 days. Mice were immunized with a dose containing 1 μg YU-gp120 protein in 100μL of PBS in the flanks.

### YU-gp120-SpyTag HIV envelope production

A SpyTag sequence was inserted in the N-terminal part of the YU-gp120 sequence. The recombinant protein was produced in 293F cells transfected with YU-gp120-Spytag expressing pcDNA3.1 plasmid (a kind gift from Barton Haynes) as described in^61^. Briefly, cell supernatant was filtered with 0.8 μm filter, mixed with *Galanthus nivalis* lectin (GNL) binding buffer and loaded onto GNL-agarose column previously equilibrated with GNL binding buffer (5 times the volume). After that, the column was washed 5 times with GNL binding buffer and the protein was eluted using a Mannose solution. The purity was assessed by running an SDS-PAGE.

### CR2-C3dg binding measurement

The on- and off-rate and the equilibrium dissociation constant for the CR2 interaction with C3dg was measured using bio-layer interferometry (Octet, Sartorius). We loaded a his-tagged human CR2 protein (Bio-techne) to Nickel-NTA sensor at a concentration of 36.8 nM. Human C3dg protein was produced as described^62^. Briefly, human C3dg cDNA containing the Cys1010Ala mutation inserted into pET13b expression plasmid lacking the his-tag (a kind gift from J. Eisenman) was transformed into BL21 E. coli. After induction with IPTG, soluble C3dg was purified from bacterial lysates using CM Sepharose followed by gel filtration on a Superdex 200 column. Association of the C3dg protein with CR2 was measured for concentrations ranging from 0.023 μM to 2.9 μM. The equilibrium dissociation constant was determined by fitting the plateau values with a binding model, yielding KD = 317 ± 30 nM. The association and dissociation rates were determined by fitting a kinetic model yielding KON = 616,154 M^-1^ s^-1^, KOFF = 0.15 s^-1^.

### IC-FDC dissociation mathematical model

To describe the dynamics of IC dissociation from FDCs at varying CR2 concentrations, we generated a stochastic framework comprising microscopic events that alter the probability of IC survival over time. Specifically, as an IC is loaded onto the FDC membrane, a multiplicity of adhesive bonds forms between the C3d ligands coating the IC particle and the surface CR2. Individual dissociated CR2-C3d pairs can rebind, as long as some bonds remain to hold the IC. Once all bonds open, the IC is irreversibly lost.

Mathematically, starting from maximum bond formation between the IC and CR2 on the corresponding membrane patch (assuming that each membrane patch can host at most one IC), stochastic IC loss proceeds through a one-step master equation:

$$\frac{dP_{m}}{dt}=r(m+1)P_{m+1}(t)+g(m-1)P_{m-1}(t)-(r(m)+g(m))P_{m}(t).$$

Here *P_m_*(*t*) represents the probability that *m* bonds remain closed between an IC and the FDC at time *t*, which evolves due to dissociation of any closed bond at an unbinding rate *r*(*m*) = *mk_off_* and formation of a new bond at a rebinding rate *g*(*m*) = *C(m)k_on_*. We set *g*(0) = 0 to avoid IC re-association. Rebinding shifts the equilibrium state away from complete dissociation, stabilizing multivalent binding (IC survival) in the presence of noise. Eventually, all bonds break if one waits long enough. The CR2-C3d single-bond dissociation rate, *k_off_*, was obtained from Biolayer Interferometry. In the model $k_{off}^{-1}=1/(0.15s^{-1})\approx 7s$ sets the time unit. *k_on_* was adjusted to recapitulate the typical half-life of ICs observed at known CR2 concentrations.

The key quantity in the rebinding rate, *C*(*m*), counts the number of binding configurations (bond arrangements) given *m* closed bonds. Importantly, the form of *C*(*m*) depends on bond properties (e.g. length and flexibility) and binding geometry (e.g. C3d spacing, curvature of the IC surface, distance of IC from the FDC surface when bound). We used the all-to-all binding scenario: *C*(*m*) = (*n_R_* – *m*)(*n_L_* – *m*), whereby each of *n_L_* C3d ligands on a guest IC is accessible to all *n_R_* CR2 on the host membrane patch. This scenario is appropriate for long, flexible molecules like CR2. Note that *C*(*m*) changes rapidly with the valency *m* due to nonlinearity, resulting in a high sensitivity of IC survival to CR2 concentration.

We simulated the model and computed the time-dependent survival probability of an IC, $S(t)=\sum_{m=1}^{\text{min}\;(n_{R},n_{L})}P_{m}(t)$, for a given CR2 concentration (*n_R_*). To account for variations of CR2 density for a given mean value (corresponding to different FDC populations), we consider a Poisson distribution of CR2 level among membrane patches for a certain mean CR2 density 〈*n_R_*〉: *P*(*n_R_*) = e^–〈*n_R_*〉^〈*n_R_*〉*n_R_*/*n_R_*. By averaging over this distribution, we obtain the mean IC survival rate 〈*S*(*t*))〉 and use 〈*S*(*t*))〉 · 〈*n_R_*〉 to represent the overall surface concentration of IC on the FDCs. In Figure 6B we plot this quantity against time and also extract the half-life (time taken to reach half of the initial IC level) at varying CR2 concentrations.

### anti-CD40L treated mice

Mice were immunized with a first antigen immunocomplex (Immunization protocol) and five days later injected intraperitoneally with 200 μg anti-CD40L blocking antibody or its isotype control (BioXCell) for two subsequent days. Mice were subsequently immunized with a second antigen immunocomplex and injected again two days later with 200 μg anti-CD40L blocking antibody or its isotype control for 2 subsequent days. Draining LNs were used for flow cytometry and microscopy.

### anti-CD21/35 blocking antibody treated mice

Mice were injected with 2 μg anti-CD21/35-BV421 (clone 7G6; BD Biosciences) subcutaneously in the upper and lower flank to target the brachial, axillary and inguinal draining LNs starting on the day of the experiment and every 2 days thereafter up to 3 injections. Mice were subsequently immunized with antigen-immunocomplexes (following the immunization protocol described above) and analyzed after 24, 4 and 7 days postimmunization.?

### ELISA

In immunized mice, sera were obtained at 21and 56 days after immunization. Plate-bound NP(7)-BSA and NP(25)-BSA (10 μg/ml) was used to measure antigen-specific antibodies. Class-switched serum immunoglobulin levels were detected using SBA Clonotyping System HRP kit (Southern Biotech). Antibody titers are given as -log2(dilution) x 40. Positive values were defined as those 3 s.d above mean values of the negative controls^6^.

### Quantification and statistical analysis

Prism v9.1.2 (GraphPad Software) was used to assess statistical significance of non-RNA-seq data. The sample size (*n*), statistical significance and statistical tests are indicated in the legends. Data distribution was assumed to be normal, but this was not formally tested. Data collection and analysis were not performed blind to the conditions of the experiments and no formal randomization was used. No statistical methods were used to pre-determine sample sizes but our sample sizes are similar to those reported in previous publications^6^. All data points were analyzed unless there were technical errors.

## Extended Data

## Data Availability

Ensembl GRCm38 was used as the reference genome to build the index. The mouse scRNAseq data are available in GEO under accession no GSE213254.

## Code Availability

Matlab script for FDC segmentation is available at https://github.com/ptolar/FDCseqmentation. Code used to analyze scRNAseq is available at https://github.com/FrancisCrickInstitute/Cxcl13_project.
